# Sonodynamic and Bioorthogonal Sonocatalytic Thrombotic Therapy Based on AIE Cationic Tetranuclear Ir(III) Complex Nanoplatform Guided by NIR‐Chemiluminescence Imaging

**DOI:** 10.1002/adma.202503599

**Published:** 2025-07-10

**Authors:** Zihan Wu, Liping Zhang, Ziwei Wang, Shengnan Liu, Qiaohua Zhang, Chunguang Shi, Yachao Wang, Gelin Xu, Dongxia Zhu, Martin R. Bryce, Lijie Ren, Ben Zhong Tang

**Affiliations:** ^1^ Key Laboratory of Nanobiosensing and Nanobioanalysis at Universities of Jilin Province Department of Chemistry Northeast Normal University 5268 Renmin Street Changchun Jilin Province 130024 China; ^2^ Department of Neurology Inst Translat Med The First Affiliated Hospital of Shenzhen University Shenzhen Second People's Hospital Shenzhen 518035 China; ^3^ Department of Chemistry Durham University Durham UK; ^4^ School of Science and Engineering Shenzhen Institute of Aggregate Science and Technology The Chinese University of Hong Kong (CUHK‐Shenzhen) Shenzhen Guangdong 518172 China

**Keywords:** aggregation‐induced emission, bioorthogonal catalysis, iridium complex, near‐infrared chemiluminescence, sonodynamic therapy

## Abstract

Non‐invasive thrombolytic strategies include photothermal therapy (PTT), photodynamic therapy (PDT) and sonodynamic therapy (SDT). The development of PTT and PDT is impeded by the limited tissue penetration depth of the light source. SDT is a promising method due to the deeper tissue penetration and site‐specific features of ultrasound; however, the scarcity of efficient sonosensitizers has constrained its clinical use. Herein, ultrasound‐triggered nanoparticles (NPs), named Ir‐4@S‐R NPs, achieve a sonodynamic thrombolytic, bioorthogonal sonocatalytic reaction, with chemiluminescence (CL) imaging in the thrombus. An Ir(III) complex, named Ir‐4, which has aggregation induced emission (AIE), combined with a hydrogen sulfide donor, named H_2_S‐N_3_, provide the nanoplatform which is modified with the peptide c(RGDfC) to target the thrombus. This supramolecular assembly successfully integrates: i) thrombosis detection based on near‐infrared (NIR) chemiluminescence imaging, ii) sonodynamic thrombus therapy and iii) release of hydrogen sulfide for anti‐inflammatory action. Ir‐4@S‐R NPs emit long‐lasting NIR CL triggered by endogenous ONOO^−^ and achieve 12 mm deep tissue penetration. Blood clots are effectively removed and blood flow is almost fully restored in mouse carotid thrombus and rat femoral vein models. The work demonstrates a new integrated strategy for diagnosing and treating life‐threatening diseases caused by thrombusotic disorders.

## Graphical abstract

AIE‐active tetranuclear Ir(III) complex Ir‐4 as a sonosensitizer/sonocatalyst is formulated into a nanoplatform with an H_2_S donor. Ir‐4@S‐R NPs achieves combined endogenous NIR chemiluminescence imaging, sonodynamic therapy and release of H_2_S in situ by a bioorthogonal sonocatalytic reaction in vivo, achieving safe thrombolysis and anti‐inflammatory therapy.

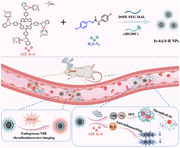

## 1 Introduction

The genesis of a thrombus (a blood clot) within the vasculature is the main culprit of numerous critical cardiovascular pathologies which constitute the foremost cause of global morbidity and mortality for humanity.^[^ ^1^, ^2^, ^3^ ^]^ Current treatments for thrombosis mainly involve drug therapy^[^ ^4^ ^]^ and surgery.^[^ ^5^ ^]^ However, these treatments have limitations and drawbacks. Surgery is effective but invasive and risky. Drug therapies are in rapid development, but they often cause bleeding complications, have short half‐lives, and lack specificity.^[^ ^6^, ^7^ ^]^ Moreover, thrombus formation and dissolution can trigger inflammatory factors that serve as key mediators to further activate the aggregation of platelets, thereby expanding the thrombus.^[^ ^8^, ^9^, ^10^ ^]^ The functional interdependence of these processes has become increasingly well defined.^[^ ^11^ ^]^ Therefore, there is an urgent need to develop a comprehensive thrombus treatment to achieve precision therapy and effective mitigation of inflammatory damage.

Sonodynamic therapy (SDT) is a promising method for thrombus treatment due to the non‐invasive, deep‐tissue penetration and site specificity of ultrasound (US).^[^ ^12^, ^13^ ^]^ However, local US energy alone may not be sufficient to effectively dissolve the thrombus without the integration of thrombolytic drugs. Sonosensitizers are activated by US, facilitating transition from their ground state to an excited state. Sonosensitizers typically undergo intersystem crossing (ISC) to a longer‐lived triplet state, resulting in the conversion of ground‐triplet‐state ^3^O_2_ into excited‐singlet‐state ^1^O_2_. Alternatively, the US‐activated sonosensitizers interact directly with biological substrates, acquiring a hydrogen atom or an electron, thereby generating free radicals.^[^ ^14^ ^]^ Reactive oxygen species (ROS) can disrupt the phospholipids and fibrin skeleton to dissolve the thrombus.^[^ ^15^ ^]^ The enhanced spin‐orbit coupling favored by the heavy atom effect in sonosensitizers (such as iodine or iridium) can also increase the ISC rate, thereby promoting the generation of ROS.^[^ ^16^, ^17^, ^18^, ^19^ ^]^ Low aqueous solubility of sonosensitizers leads to reduced ROS generation due to aggregation‐caused quenching (ACQ).^[^ ^20^, ^21^ ^]^ However, the opposite phenomenon of aggregation‐induced emission (AIE) can enhance fluorescence and ROS generation in an aggregate state through restriction of intramolecular motions, thereby reducing the dissipation of energy.^[^ ^22^, ^23^, ^24^ ^]^ Ir(III) complexes are a new class of sonosensitizer due to their excellent ROS production, high sonostability and tunable ligand modification.^[^ ^16^ ^]^ However, ultrasound therapy using Ir(III) complexes is still in its infancy, and to date AIE Ir(III) complexes have not been reported as sonosensitizers for biological applications.

Our group reported a series of multinuclear AIE Ir(III) complexes utilizing Schiff bases as both chelating and bridging motifs which possess excellent ROS production capacity.^[^ ^25^, ^26^ ^]^ Nevertheless, the excitation light source of traditional Ir(III) complexes is mainly in the blue and ultraviolet (UV) regions, which imposes a serious limitation of tissue penetration depth and thus obstructs their biological applications. AIE Ir(III) complexes with near‐infrared (NIR) excitation have only seldom been obtained^[^ ^27^, ^28^ ^]^ mainly due to the fast nonradiative deactivation process via intramolecular vibrational relaxations known as the “energy gap law.”^[^ ^29^ ^]^ Given these circumstances, we introduced photon upconversion nanoparticles to overcome the penetration depth limitation.^[^ ^26^ ^]^ Nevertheless, low energy conversion efficiency seriously hindered their therapeutic effect. Hence, how to obtain a sonosensitizer based on an AIE Ir(III) complex is of great significance to solve the critical issue of inadequate ROS generation in sonodynamic therapy.

Hydrogen sulfide (H_2_S) plays an important role in the treatment of vascular diseases by regulating vascular tension, inhibiting vascular inflammation^[^ ^30^ ^]^ and facilitating vascular cell apoptosis.^[^ ^31a,b^ ^]^ The utilization of H_2_S in this context has been constrained by its short half‐life, narrow therapeutic window and high reactivity.^[^ ^32^ ^]^ Bioorthogonal chemistry offers “new‐to‐nature” reactions for the on‐site generation of therapeutic agents.^[^ ^33^, ^34^ ^]^ Consequently, bioorthogonal catalysis provides a new strategy for the delivery of H_2_S. In 2023 Wang et al reported the self‐assembly of proteins with hydrogen‐bonded organic frameworks (HOFs) that acted as bioorthogonal catalysts to generate H_2_S; this is the only report until now.^[^ ^35^ ^]^ The work provides a versatile set of chemical tools for mitochondria‐targeted bioorthogonal catalytic prodrug activation. However, potentially due to the limited penetration depth of the short‐wavelength excitation light source (470 nm), Wang's research was confined to in vitro cellular studies.^[^ ^35^ ^]^ Pioneering works have reported bioorthogonal photocatalysis based on iridium complexes.^[^ ^36^ ^]^ For example, Fan and Chen et al applied Ir(III) complexes to the labeling of biological samples by bioorthogonal photocatalytic reactions.^[^ ^37^, ^38^ ^]^ However, the limited tissue penetration depth is still a critical factor seriously restricting these types of reactions. Thus, ultrasound as a form of bioorthogonal excitation energy offers an innovative approach to enhance tissue penetration. Chen et al reported a bioorthogonal catalyst based on modified copper nanocomplexes in 2022, and achieved high spatiotemporal catalytic efficacy under exogenous ultrasound irradiation.^[^ ^34^ ^]^ However, until now, there has been no report of a bioorthogonal catalyst reaction triggered by an Ir(III) complex using ultrasound.

Early identification of a thrombus is vital for efficient therapy and to decrease its recurrence.^[^ ^39^, ^40^ ^]^ Conventional techniques to diagnose and monitor thrombus disorders are magnetic resonance imaging (MRI) and computed tomography (CT).^[^ ^41^, ^42^ ^]^ However, the limited imaging sensitivity of these methodologies and the nonspecific symptoms of a thrombus impede accurate and prompt diagnosis. Fortunately, molecular imaging strategies utilizing luminescent and photoacoustic imaging probes facilitate precise location of thrombus‐associated abnormalities.^[^ ^43^, ^44^, ^45^, ^46^ ^]^ Nevertheless, these luminescent probes encounter the challenge of tissue autofluorescence, which diminishes the signal‐to‐background ratio (SBR).^[^ ^47^ ^]^ Although NIR fluorophores can mitigate tissue autofluorescence, attaining high penetration depth remains a formidable challenge due to the restricted penetration of NIR light.^[^ ^48^ ^]^ Unlike fluorescence, chemiluminescence (CL) imaging does not require an external light source. Instead, CL harnesses energy generated by in situ chemical reactions, thereby leading to minimized tissue autofluorescence and enhanced imaging sensitivity.^[^ ^49^, ^50^, ^51^, ^52^ ^]^ However, the familiar CL probes (such as luminol and peroxyoxalate) mainly emit visible light, which restricts their tissue penetration depth.^[^ ^53^, ^54^ ^]^ Some NIR CL probes employ a chemiluminescence resonance energy transfer (CRET) mechanism between the CL donor and the fluorophore acceptor.^[^ ^55^, ^56^ ^]^ Due to the complex multistep manipulation and the inherently low efficiency of intermolecular energy transfer, there is inevitable energy dissipation, and relay processes decrease the CL quantum yield.^[^ ^57^ ^]^ Therefore, the development of a single molecular architecture for NIR CL‐guided sonodynamic therapy to combat thrombus is imperative.

**Scheme 1:**
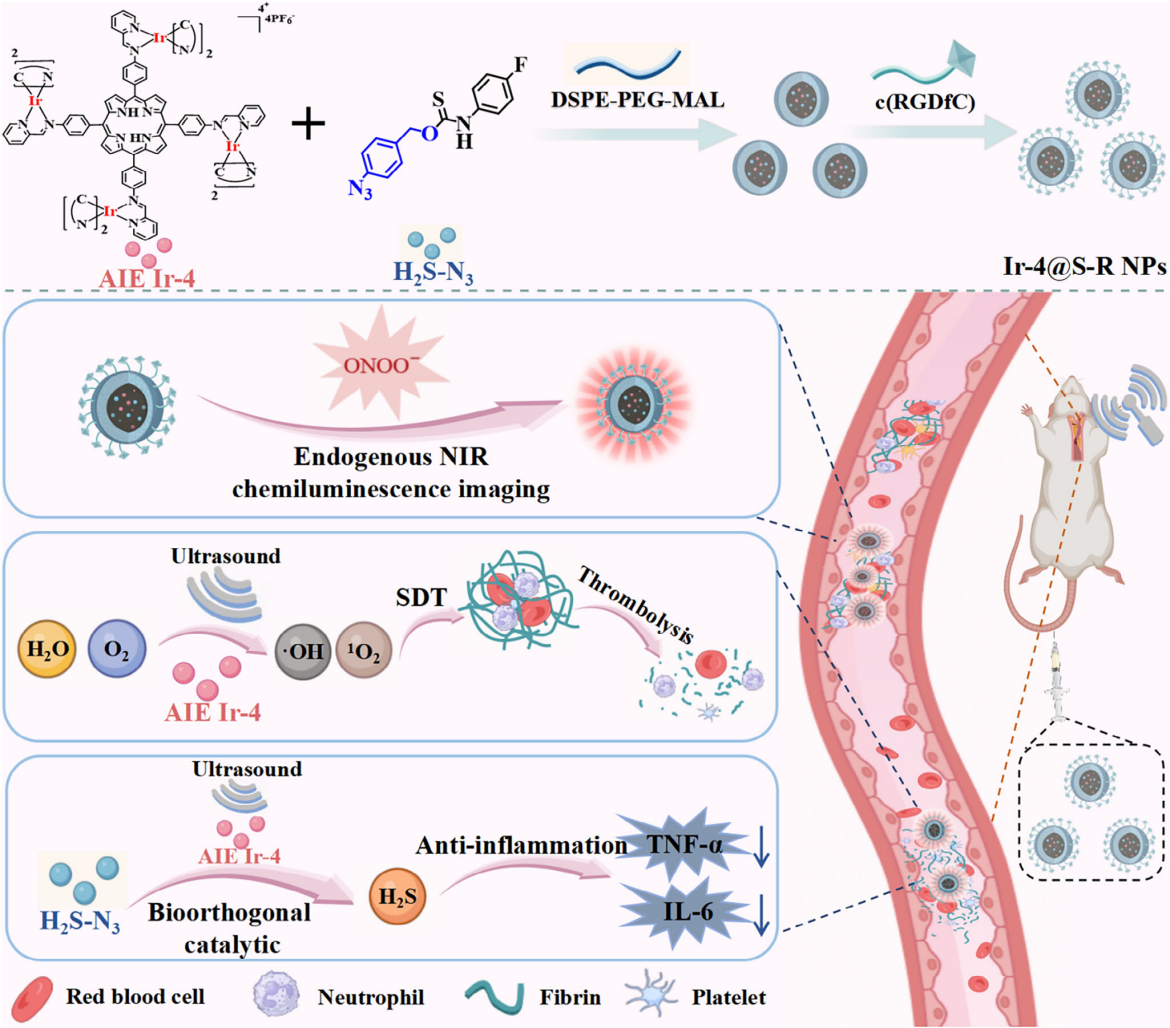
Schematic illustration of the thrombus‐specific nanoplatform Ir‐4@S‐R NPs for NIR CL imaging and ultrasound‐triggered synergistic treatment of thrombosis.

To overcome the drawbacks outlined above, we have developed for the first time a sonosensitizer/sonocatalyst nanoplatform based on an AIE‐active tetranuclear Ir(III) complex to achieve SDT thrombolysis and anti‐inflammatory therapy under endogenous ONOO^−^‐triggered NIR CL guidance (**Scheme** **1**). The Ir(III) complex and H_2_S donor were engineered into nanoparticles (NPs), namely Ir‐4@S‐R NPs, with the following features: 1) the AIE tetranuclear Ir(III) complex, named Ir‐4, demonstrates excellent ROS production; 2) the H_2_S donor comprising an aryl azide *O*‐thiocarbamate unit, named H_2_S‐N_3_, releases H_2_S in situ;^[^ ^31^, ^35^ ^]^ 3) NPs were prepared with hydrophilic DSPE‐PEG‐MAL to provide high biocompatibility; 4) the NPs were modified with the peptide c(RGDfC) to ensure the desired thrombus targeting effect. The Ir‐4@S‐R NPs represent the pioneering example of an AIE Ir(III) complex‐based sonosensitizer to achieve an excellent SDT thrombolytic effect in vitro and in vivo. This work also represents the first application of an Ir(III) complex as a bioorthogonal sonocatalyst for H_2_S release and effective anti‐inflammatory protocol. Furthermore, Ir‐4@S‐R NPs show bright and deeply tissue penetration NIR CL triggered by endogenous ONOO^−^ in a mouse carotid thrombus model.

## 2 Results and Discussion

Taking advantage of the tunable ligand structure of Ir(III) complexes, we synthesized N^N ligands that incorporate an imine bond by using a simple and high‐yielding Schiff base reaction. The introduction of strong electron‐withdrawing groups in the C^N ligands of Ir(III) complexes improves their redox capacity.^[^ ^58^ ^]^ Therefore, 2‐(2,4‐difluorophenyl)‐5‐(trifluoromethyl)pyridine was chosen as the C^N ligand in this work. Tetraphenylporphyrin (TPP) was linked to the Ir(III) complexes as chemiluminescent subunits to obtain mononuclear and tetranuclear cationic Ir(III) complexes (Ir‐1 and Ir‐4). The synthetic routes to the ligands L1, L4, and the complexes Ir‐1 and Ir‐4, rhodamine bisazide 1, and H_2_S‐N_3_ (Schemes S1–S6, Supporting Information) and corresponding characterization data are provided in the supporting information with ^1^H NMR and mass spectra (Figures S1–S8, Supporting Information).

**Figure 1:**
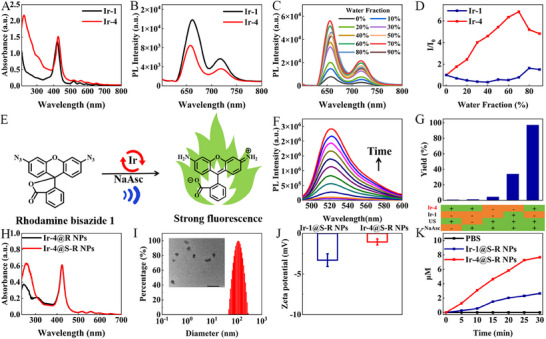
A) UV–vis absorption spectra of Ir‐1 and Ir‐4 in THF (complex concentration = 1.0 ×10^−5^ M); B) Fluorescence spectra of Ir‐1 and Ir‐4 in THF (complex concentration = 1.0 ×10^−5^ M) under 365 nm UV irradiation; C) Emission spectra of Ir‐4 in THF‐H_2_O mixtures (complex concentration = 1.0 × 10^−5^ M) with different water fractions (0%–90% v/v) at room temperature; D) The change of the emission intensity of Ir‐1and Ir‐4 in THF‐H_2_O with the change of the water concentration; E) Sonoreduction of Rhodamine bisazide 1 catalyzed by Ir‐1 or Ir‐4 under ultrasound irradiation (1.0 W cm^−2^, 1 MHz, 50% duty cycle); F) Fluorescence intensity of the reaction mixture in water at different time intervals. Ir‐4 = 5 µM, Rhodamine bisazide 1 = 40 µM, NaAsc = 2 mM; G) Rhodamine bisazide 1 reduction reaction yield in different conditions; H) The absorption spectra of Ir‐4@R NPs and Ir‐4@S‐R NPs at the same concentration; I) DLS diagram of Ir‐4@S‐R NPs, inset: the TEM image of Ir‐4@S‐R NPs. Scale bar: 200 µm; J) zeta potential of Ir‐1@S‐R NPs and Ir‐4@S‐R NPs as analyzed by DLS. Data are presented as mean ± SD (n = 3 independent experiments); K) The amount of H_2_S released from PBS, 200 µg mL^−1^ Ir‐1@S‐R NPs and 200 µg mL^−1^ Ir‐4@S‐R NPs under ultrasound irradiation (1.0 W cm^−2^, 1 MHz, 50% duty cycle).

The ultraviolet–visible (UV–vis) absorption and fluorescence spectra of Ir‐1 and Ir‐4 were studied in tetrahydrofuran (THF) solution. **Figure** **1**A shows relatively strong absorption bands in the 250–350 nm range due to the π–π* transition at the ligand center, while the bands spanning 350–500 nm are a combination of metal‐to‐ligand charge transfer (^1^MLCT), ligand‐to‐ligand charge transfer (^1^LLCT), spin‐forbidden metal‐to‐ligand charge transfer (^3^MLCT), and spin‐forbidden ligand‐to‐ligand charge transfer (^3^LLCT). The absorption bands in the 500–700 nm range are due to the porphyrin component, demonstrating that the iridium complexes with long‐wavelength absorption have been successfully constructed by incorporating a porphyrin unit. As shown in Figure 1B, Ir‐1 and Ir‐4 have a strong emission peak at about 662 and 656 nm, respectively, in the red region and a weaker peak at about 720 nm in the NIR region. However, the photoluminescent (PL) intensity significantly decreased in the order Ir‐1 > Ir‐4. The increase of absorption capacity and the decrease of emission intensity for Ir‐4 are favorable to obtain a considerably longer‐lived triplet photoexcited state.^[^ ^59^ ^]^ Figure 1C,D show that Ir‐4 exhibited weak emission in THF, but bright NIR fluorescence when the water content gradually increased to 70%, which is typical AIE behavior.^[^ ^60^ ^]^ In contrast, Ir‐1 did not show AIE (Figures 1D and S9, Supporting Information). These results indicate that the AIE‐active tetranuclear complex Ir‐4 is favorable for ROS generation. To verify the potential for SDT applications, 2′,7′‐dichlorodihydrofluorescein (DCFH) was used as a probe for ROS generation. As shown in Figure S10, Supporting Information, the emission peak of DCFH at 525 nm increases in the presence of the Ir complex upon ultrasound irradiation (1.0 W cm^−2^, 1 MHz, 50% duty cycle). The aggregated form of Ir‐4 (fw: 90%) represents better ROS generation ability (*I*/*I*_0_ ≈ 8) than free Ir‐4 (fw: 0%) (*I*/*I*_0_ ≈ 1.7), which should be attributed to the beneficial reduction in nonradiative transitions caused by aggregation‐induced restricted intramolecular motion (RIM).^[^ ^22^ ^]^ Transmission electron microscopy (TEM) images in Figure S11, Supporting Information show that Ir‐4 exhibits spherical particles of ≈100 µm diameter at 90% water fraction, while Ir‐1 does not aggregate under these conditions. In addition, the fluorescence quantum yield of Ir‐4 was enhanced in the aggregated state as shown in Table S1, Supporting Information. These results further demonstrate that Ir‐4 has the potential for SDT due to the better ROS generation ability originating from the AIE performance.

We first explored the sonocatalytic reduction potential of Ir‐1 and Ir‐4 in aqueous solution, using azide‐caged Rhodamine 110 as the substrate. The reduction of the azide units in the fluorogenic Rhodamine bisazide 1 into amines led to an enhancement in fluorescence, serving as an indicator for evaluating the catalytic efficacy (Figure 1E). In reaction mixtures containing 40 µM of Rhodamine bisazide 1, 2 mM sodium ascorbate (NaAsc), and 5 µM Ir‐4, a marked increase in fluorescence was observed upon ultrasound irradiation (1.0 W cm^−2^, 1 MHz, 50% duty cycle) (Figure 1F). This clearly demonstrates the high catalytic proficiency and rapid kinetics associated with Ir‐4 in the sonocatalytic reduction of azides. The reaction achieved a conversion rate exceeding 95%, as determined by the fluorescence intensity of the resultant mixture against a standard of pure Rhodamine 110 (Figure 1G). In contrast, negative controls with either Ir‐1, or missing one of the reaction conditions (NaAsc, ultrasound irradiation, or Ir‐4), showed only minor changes in fluorescence intensity (Figures S12 and S13, Supporting Information). The above data illustrate that Ir‐4 exhibits good sonocatalytic ability. We then explored the tissue penetration ability of ultrasound. The fluorescence signal from the reduction of Rhodamine bisazide 1 was analyzed when the reaction mixture was covered with chicken breast tissue of thickness 2, 4, and 6 mm under ultrasound irradiation (1.0 W cm^−2^, 1 MHz, 50% duty cycle) or under 635 nm laser irradiation (0.8 W cm^−2^). Figure S14, Supporting Information shows that the fluorescence of the laser group is slightly stronger than the ultrasound group covered with 2 mm chicken breast. However, when covered with 4 mm thick chicken breasts, the fluorescence of the laser group dropped sharply, whereas in the ultrasound group the fluorescence decreased by only about 30% compared with that of 2 mm thickness. For 6 mm thickness, there was almost no change in fluorescence in the laser group, indicating that the laser could not excite the iridium complex in deep tissue to release Rhodamine fluorescence. In contrast, the fluorescence signal was significantly enhanced with time in the 6 mm ultrasound experiment, showing that ultrasonic energy can be effectively delivered into deep tissue. Hence, ultrasound can replace a laser as an excellent irradiation source to excite transition metal complexes for bioorthogonal sonocatalytic reactions.

Ir‐1 or Ir‐4 and H_2_S‐N_3_ (H_2_S donor)^[^ ^35^ ^]^ were assembled into water‐soluble nanoparticles (NPs) named Ir‐1@S NPs or Ir‐4@S NPs via a one‐step nanoprecipitation method with DSPE‐PEG‐MAL as the surfactant. To endow thrombus‐homing ability, the peptide c(RGDfC) was attached to the NPs to give Ir‐1@S‐R NPs or Ir‐4@S‐R NPs (Figure S15, Supporting Information). As illustrated in Figure S16, Supporting Information, the characteristic azide peak of H_2_S‐N_3_ appeared in the FT‐IR spectra of Ir‐1@S‐R NPs and Ir‐4@S‐R NPs. The UV–vis‐NIR absorption spectrum of Ir‐1@S‐R NPs and Ir‐4@S‐R NPs displayed a maximum at ≈420 nm, which is consistent with that of Ir‐1 and Ir‐4. In addition, a new absorption peak at 255 nm was attributed to H_2_S‐N_3_ (Figure 1H, Figure S17, Supporting Information). These data indicate that the H_2_S donor was successfully introduced into Ir‐1@S‐R NPs and Ir‐4@S‐R NPs. The micro‐morphology and size of the NPs were determined by dynamic light scattering (DLS) and transmission electron microscopy (TEM). The average hydrodynamic diameters of Ir‐1@S‐R NPs and Ir‐4@S‐R NPs are 90 nm and 113 nm (Figures 1I and S18, Supporting Information). Ir‐1@S‐R NPs and Ir‐4@S‐R NPs both have a uniform spherical shape, and the average size is 78 nm and 95 nm by TEM (Figure 1I inset and Figure S15, Supporting Information inset). Upon the modification with c(RGDfC) peptide, the zeta potential of Ir‐1@S‐R NPs and Ir‐4@S‐R NPs was −3.3 and −1.1 mV, respectively. The diameters of all of the NPs remained almost unchanged after 7 days in water, indicating their robust stability (Figure S19, Supporting Information). According to the absorption spectra of H_2_S‐N_3_ standard solutions in THF, the encapsulation efficiency of H_2_S‐N_3_ in Ir‐1@S‐R NPs and Ir‐4@S‐R NPs was 42.2% and 29.1% (Figure 1H, Figures S14 and S20, Supporting Information).

The ultrasound‐triggered release of H_2_S from both Ir‐1@S‐R NPs and Ir‐4@S‐R NPs increased with time (Figures 1K and S21, Supporting Information). However, about fourfold more H_2_S was released after 30 min for Ir‐4@S‐R NPs compared with Ir‐1@S‐R NPs. Moreover, the H_2_S generation from Ir‐4@S‐R NPs was dose‐dependent: more H_2_S was detected with increasing the concentration of Ir‐4@S‐R NPs (Figure S22, Supporting Information). The above results demonstrate the first bioorthogonal sonocatalytic nanoplatform based on an AIE Ir(III) complex to achieve efficient H_2_S delivery in situ.

**Figure 2:**
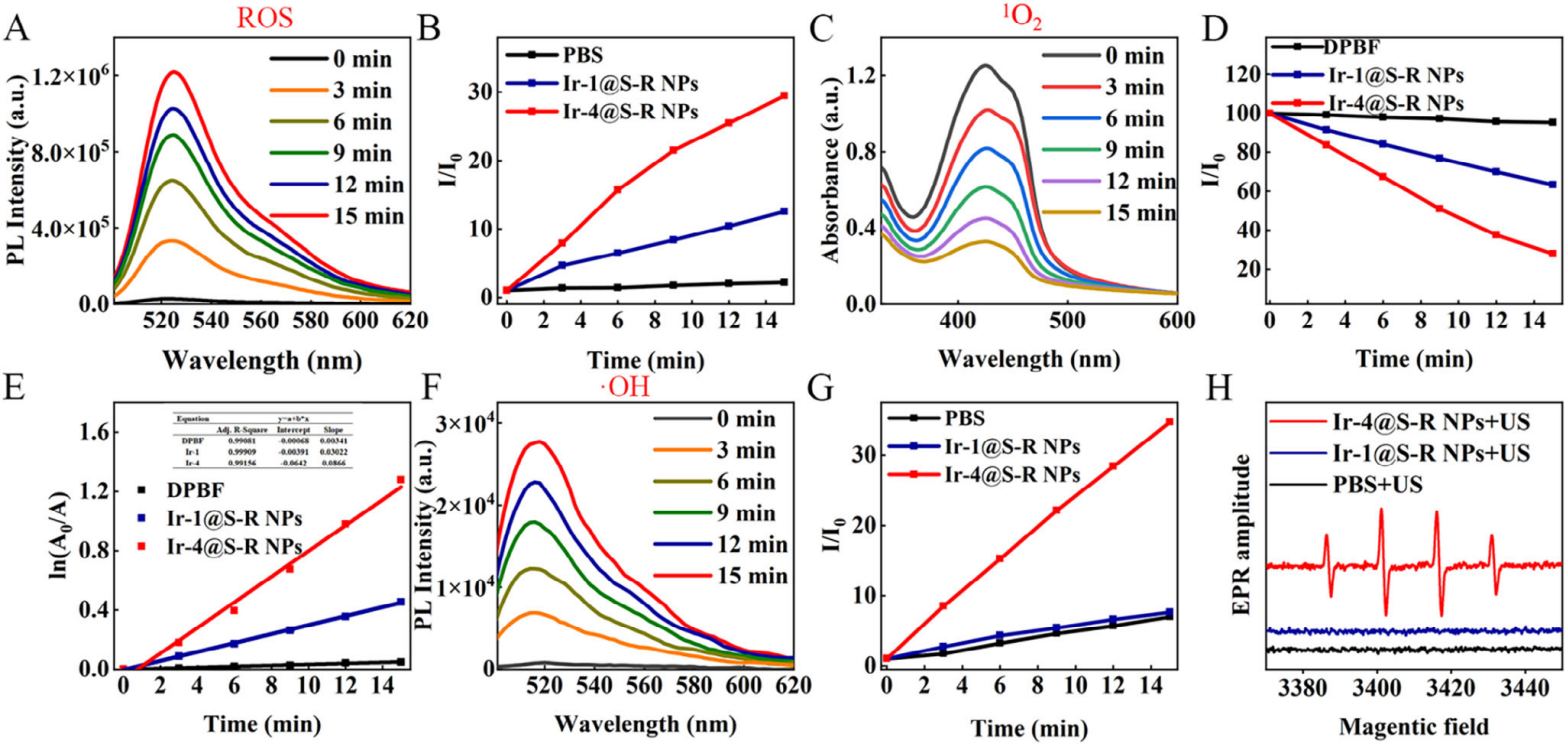
A) PL spectra changes of DCFH in the presence of Ir‐4@S‐R NPs under ultrasound irradiation (1.0 W cm^−2^, 1 MHz, 50% duty cycle); B) The PL intensity of DCFH with time under different conditions. I_0_ = initial intensity at 525 nm. I = real‐time intensity at 525 nm with various times of ultrasound irradiation; C) UV–vis absorption spectral changes of DPBF in the presence of Ir‐4@S‐R NPs under ultrasound irradiation (1.0 W cm^−2^, 1 MHz, 50% duty cycle); D) Comparison of the decay rates at different conditions under ultrasound irradiation (1.0 W cm^−2^, 1 MHz, 50% duty cycle), I_0_ = absorption of DPBF without irradiation. I = real‐time absorption of DPBF with different irradiation time; E) Time‐dependent ^1^O_2_ generation kinetics. A_0_ = absorption of DPBF without irradiation. A = real‐time absorption of DPBF with different irradiation time; F) PL spectra changes of HPF in the presence of Ir‐4@S‐R NPs under ultrasound irradiation (1.0 W cm^−2^, 1 MHz, 50% duty cycle); G) The change of PL intensity of HPF with the change of time under different conditions. I_0_ = initial intensity of 515 nm. I = real‐time intensity of 515 nm with various times under ultrasound irradiation; H) EPR signals of DMPO (for type‐I ROS detection) in the presence of PBS, Ir‐1@S‐R NPs and Ir‐4@S‐R NPs under ultrasound irradiation (1.0 W cm^−2^, 1 MHz, 50% duty cycle).

The ROS generation capacity of Ir‐4@S‐R NPs, which is important for thrombus therapy, was investigated with DCFH as the indicator upon ultrasound irradiation (1.0 W cm^−2^, 1 MHz, 50% duty cycle) in water. The emission peaks of DCFH at 525 nm in the presence of Ir‐1@S‐R NPs and Ir‐4@S‐R NPs increased over time (**Figures** **2**A and S23, Supporting Information). After 15 min irradiation with Ir‐4@S‐R NPs the intensity was > three times that with Ir‐1@S‐R NPs which indicated enhanced sonoirradiated ROS generation from Ir‐4@S‐R NPs (Figure 2B). In addition, 1,3‐diphenylisobenzofuran (DPBF) and hydroxyphenyl fluorescein (HPF) were used to distinguish the ROS types. In Figure 2C,D, the absorption band of DPBF at 410 nm decreased by >70% in the presence of Ir‐4@S‐R NPs under ultrasound irradiation within 15 min; however, this absorption decreased by only about 40% in the presence of Ir‐1@S‐R NPs. This data implies the excellent ^1^O_2_ generation ability of the Ir‐4@S‐R NPs (Figure S24, Supporting Information). In the absence of ultrasound, the absorption of the DPBF solutions of Ir‐1@S‐R NPs and Ir‐4@S‐R NPs barely decreased (Figure S24, Supporting Information) which proved that Ir‐4@S‐R NPs are an excellent sonodynamic nanoplatform. As shown in Figure 2E, ^1^O_2_ generation of Ir‐1@S‐R NPs and Ir‐4@S‐R NPs conform to first‐order kinetics. The slope follows the order: Ir‐1@S‐R NPs (0.03022) < Ir‐4@S‐R NPs (0.0866). A steeper slope indicates a greater ability to generate ^1^O_2_. As anticipated, this trend is in keeping with the number of metal centers in the sonosensitizer. According to Figure 2F, using HPF as a probe, the emission peaks of HPF at 525 nm in the presence of Ir‐4@S‐R NPs upon ultrasound irradiation increased over time. This illustrates that Ir‐4@S‐R NPs produce more •OH under ultrasound irradiation compared with the control group and Ir‐1@S‐R NPs (Figures 2G and S25, Supporting Information). Furthermore, electron paramagnetic resonance (EPR) spectroscopy probed •OH generation of the Ir‐4@S‐R NPs. 5,5‐Dimethyl‐1‐pyrroline‐*N*‐oxide (DMPO) was used as a spin‐trap to quantify the type‐I ROS. The data (Figure 2H) confirm that Ir‐4@S‐R NPs are excellent producers of •OH compared with the control group and Ir‐1@S‐R NPs upon ultrasound irradiation (1.0 W cm^−2^, 1 MHz, 50% duty cycle). The excellent ROS generation ability of Ir‐4@S‐R NPs illustrate that AIE properties and the tetranuclear Ir(III) complex structure are important preconditions to obtain highly effective sonosensitizers.

The thermal effect of ultrasound was assessed. Figure S26, Supporting Information shows the thermal effects of different volumes of PBS, Ir‐1@S‐R NPs and Ir‐4@S‐R NPs aqueous solutions under ultrasonic stimulation. The temperature increased drastically by 27.4, 28.9, and 31.1 °C within 10 min in 1 mL volume. In 10 mL volume, all three solutions rose gently by about 14 °C to about 39 °C after 10 min of ultrasonic irradiation (1.0 W cm^−2^, 1 MHz, 50% duty cycle).

**Figure 3:**
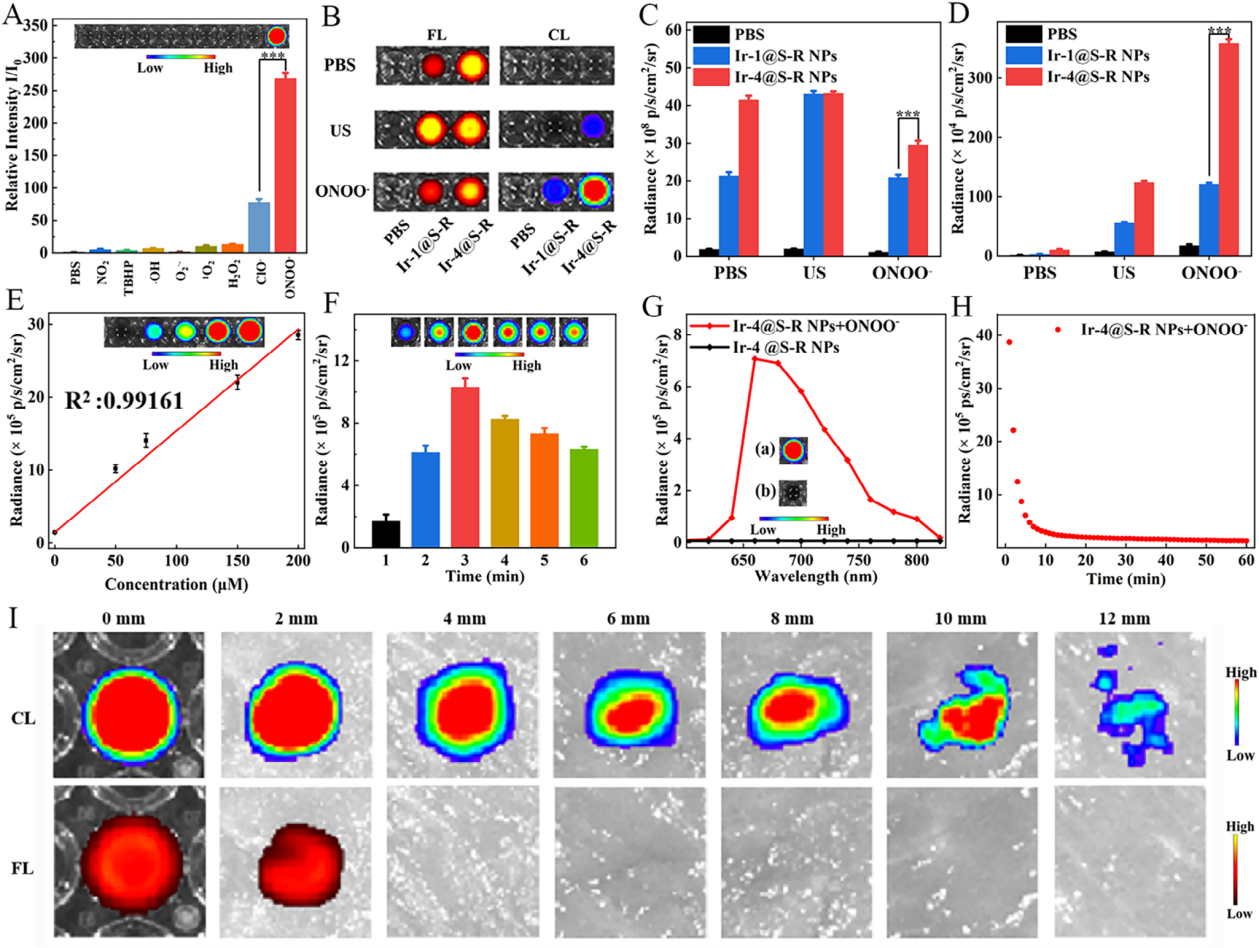
A) Chemiluminescence images of Ir‐4@S‐R NPs after addition of PBS and different RONS (200 µM for each). Data are presented as mean ± SD (n = 3 independent experiments). Inset: the chemiluminescence images acquired by an IVIS imaging system under bioluminescence mode with an open filter; B) Fluorescence and chemiluminescence images of PBS, Ir‐1@S‐R NPs (50 µM) and Ir‐4@S‐R NPs (50 µM) after cessation of the addition of PBS, ultrasound irradiation (1.0 W cm^−2^, 1 MHz, 50% duty cycle, 3 min) or the addition of ONOO^−^ (200 µM); C)‐D) The quantification of corresponding fluorescence and chemiluminescence intensities in B). Data are presented as mean ± SD (n = 3 independent experiments); E) Chemiluminescence intensities of Ir‐4@S‐R NPs after addition of ONOO^−^. Data are presented as mean ± SD (n = 3 independent experiments). Insets: the corresponding chemiluminescence acquired on an IVIS imaging system; F) Quantitative analysis of the persistent signals of the Ir‐4@S‐R NPs after ultrasound irradiation (1.0 W cm^−2^, 1 MHz, 50% duty cycle). Data are presented as mean ± SD (n = 3 independent experiments). Insets: the corresponding chemiluminescence acquired on an IVIS imaging system; G) Chemiluminescence spectra of Ir‐4@S‐R NPs in the absence or presence of ONOO^−^ (200 µM). Insets: the corresponding chemiluminescence acquired on an IVIS imaging system; H) Decay of chemiluminescence signal of Ir‐4@S‐R NPs over time at room temperature after addition of ONOO^−^; I) Tissue penetration depths after the activated chemiluminescence of NPs + ONOO^−^ and the NIR fluorescence from Ir‐4@S‐R NPs with a coverage of chicken breast tissues with different thicknesses. Data are represented as mean ± SD (n = 3). The data were analyzed by unpaired 2‐tailed Student's *t*‐test using GraphPad Prism 7 (A, C and D). *p < 0.05, **p < 0.01, ***p < 0.001.

Chemiluminescence (CL) can eliminate tissue autofluorescence noise and circumvent photon scattering of the excitation light, which is beneficial for deep tissue imaging and SBR for in vivo imaging. Ir‐4@S‐R NPs were chosen to evaluate the CL selectivity toward phosphate‐buffered saline (PBS) and reactive oxygen and nitrogen species (RONS) including NO_2_^−^, *t*‐Bu‐hydroperoxide (TBHP), OH^−^, O_2_^−^, ^1^O_2_, H_2_O_2_, ClO^−^ and ONOO^−^. Interestingly, ONOO^−^ was the most efficient initiator for Ir‐4@S‐R NPs CL; its activated CL intensity is up to 300‐fold higher than PBS or other RONS and fourfold higher than ClO^−^ (**Figure** **3**A). As shown in Figure 3B,C, in the PBS samples the Ir‐4@S‐R NPs FL was much stronger than the Ir‐1@S‐R NPs FL, which is opposite to the fluorescence results in Figure 1B for Ir‐1 and Ir‐4. This is because AIE of Ir‐4 in the NPs leads to enhanced fluorescence. The Ir‐4@S‐R NPs FL activated by ultrasound was similar to the Ir‐4@S‐R NPs FL in the PBS sample. This result indicates that Ir‐4@S‐R NPs mainly use ultrasound to generate ROS and chemiluminescence. The Ir‐1@S‐R NPs FL signal activated by ultrasound was significantly enhanced compared with the Ir‐1@S‐R NPs FL signal in the PBS sample. This result indicates that Ir‐1@S‐R NPs use ultrasound energy mainly to emit fluorescence, while the ability of Ir‐1@S‐R NPs to produce ROS might be decreased which is consistent with the previous ROS experiment results. The FL signal of Ir‐4@S‐R NPs activated with ONOO^−^ was significantly reduced compared with the Ir‐4@S‐R NPs FL signal in the PBS sample, while the FL signal of Ir‐1@S‐R NPs activated with ONOO^−^ was almost constant. This demonstrates that Ir‐4@S‐R NPs are more reactive than Ir‐1@S‐R NPs toward ONOO^−^. In Figure 3B,D, Ir‐1@S‐R NPs and Ir‐4@S‐R NPs activated with ultrasound pre‐irradiation and with ONOO^−^ both generate a CL signal, but the CL of Ir‐4@S‐R NPs is much stronger than that of Ir‐1@S‐R NPs. These results further demonstrate that ONOO^−^ can induce CL of porphyrin derivatives.^[^ ^61^ ^]^ The CL of Ir‐4@S‐R NPs activated with ONOO^−^ is four times stronger than that of Ir‐1@S‐R NPs in the same conditions. This might be because the porphyrin derivatives more readily react with substances of different electronegativity (such as ONOO^−^) after coordination of the transition metal, and the charge of Ir‐4@S‐R NPs (+4) is much higher than that of Ir‐1@S‐R NPs (+1).^[^ ^62^, ^63^ ^]^ The CL signal of Ir‐4@S‐R NPs showed a very good linear correlation with ONOO^−^ concentration, including a limit of detection (LOD) of 87.4 nM, calculated from 3σ/k (σ is the standard deviation of blank tests; k is the slope of the working curve) (Figure 3E). The CL signal of Ir‐4@S‐R NPs was enhanced with the increase of ultrasonic irradiation time, then decreased gradually after reaching a maximum value at 3 min (Figure 3F). The CL peak of Ir‐4@S‐R NPs was at ≈ 660 nm demonstrating bright NIR CL triggered by ONOO^−^ (Figure 3G). Importantly, the CL is long‐lasting (for > 10 min), which is suitable for in vivo imaging (Figure 3H, Figure S27, Supporting Information).

The imaging depth and sensitivity of the Ir‐4@S‐R NPs were studied by increasing the thickness of chicken tissue pieces placed on top of the samples, and CL was then induced by the addition of ONOO^−^. In the absence of chicken tissue, the CL image shows very low background noise and a superior SBR of 230.97 which is >13‐fold higher than that of the FL (SBR of 16.95). Both the CL and FL signals of Ir‐4@S‐R NPs decreased with increasing thickness of the chicken tissue (Figure 3I). Remarkably, a CL signal was clearly detectable at a thickness of 4 mm while the FL signal became invisible due to the poor penetration ability of the excitation light and the high background interference. As CL possesses a very low background noise, its SBR is as high as 12.15 at 12 mm thickness, whereas the FL of Ir‐4@S‐R NPs is close to the tissue autofluorescence background (Figures 3I and S28, Supporting Information). These results prove that Ir‐4@S‐R NPs exhibited excellent ONOO^−^‐triggered NIR CL imaging in deep tissue.

**Figure 4:**
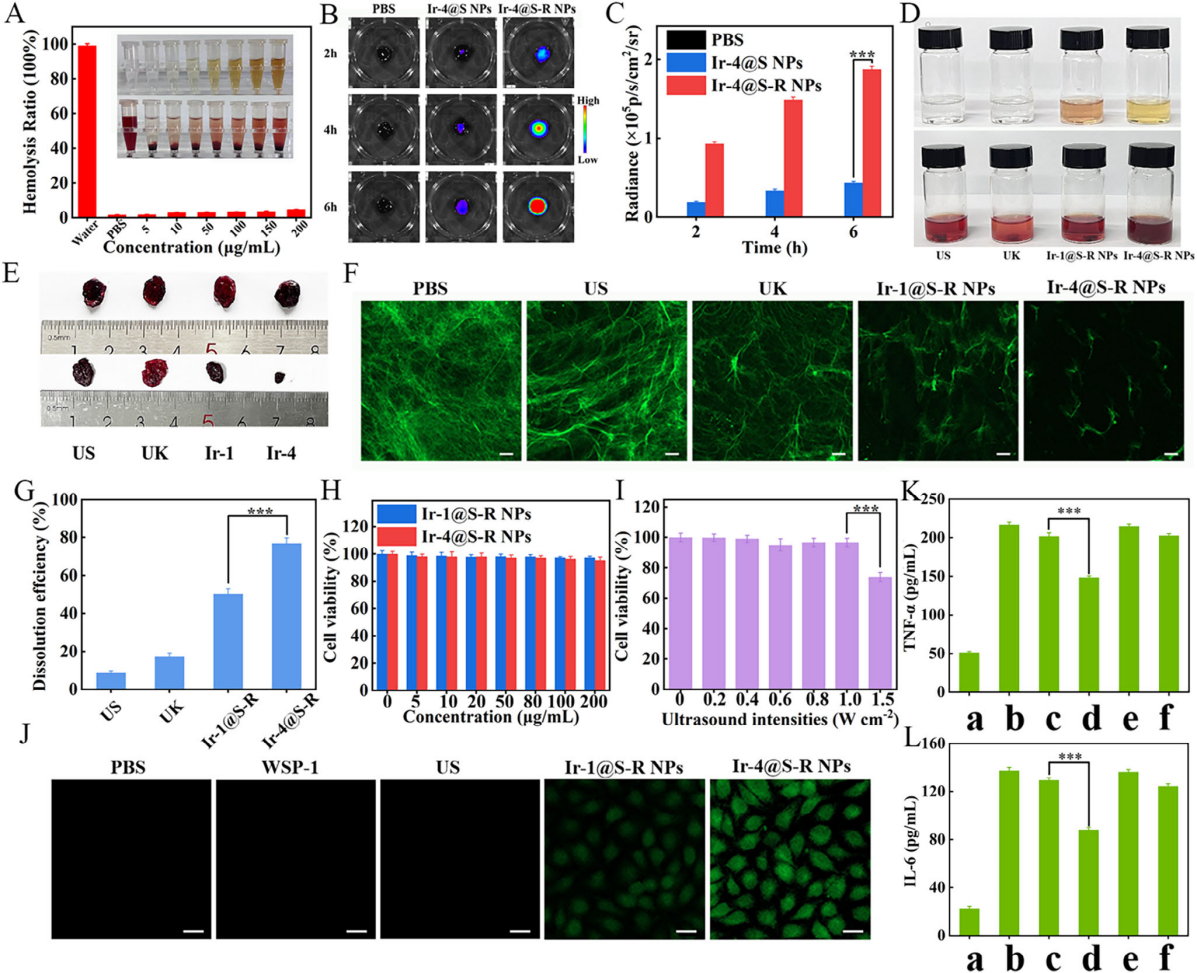
A) The hemolysis ratio of red blood cells treated with water, PBS and different concentrations of Ir‐4@S‐R NPs in water. Inset shows the red blood cells solution upon different treatments (from left to right: water, PBS, 5, 10, 50, 100, 150, and 200 µg mL^‒1^ of Ir‐4@S‐R NPs). Data are presented as mean ± SD (n = 3 independent experiments); B) The chemiluminescence images of artificial blood clots after incubation with PBS, Ir‐4@S NPs, or Ir‐4@S‐R NPs; C) Quantitative analysis of the relative persistent intensity of the artificial blood clots in B). Data are presented as mean ± SD (n = 3 independent experiments). *p < 0.05, **p < 0.01, and ***p < 0.001 versus control group; D)‐E) Photographs of the blood clot solution after different treatments (from left to right: ultrasound, free UK, Ir‐1@S‐R NPs + ultrasound, Ir‐4@S‐R NPs + ultrasound). The residual blood clots were taken out and shown; F) Fluorescence images of fibrin clots with different treatments. Scale bar: 100 µm; G) thrombolytic rates were quantitatively analyzed by measuring the percentage of thrombus mass loss after different treatments in E). Data are presented as mean ± SD (n = 3 independent experiments). *p < 0.05, **p < 0.01, and ***p < 0.001 versus control group; H) The cytotoxicity of different concentrations of Ir‐4@S‐R NPs in HUVECs. Data are presented as mean ± SD (n = 3 independent experiments). *p < 0.05, **p < 0.01, and ***p < 0.001 versus control group; I) The cytotoxicity of 10 min of ultrasound at different intensities in HUVECs. Data are presented as mean ± SD (n = 3 independent experiments). *p < 0.05, **p < 0.01, and ***p < 0.001 versus control group; J) Intracellular fluorescence imaging of H_2_S release in different treatment groups (PBS + WSP‐1, WSP‐1, ultrasound + WSP‐1, Ir‐1@S‐R NPs + ultrasound + WSP‐1, Ir‐4@S‐R NPs + ultrasound + WSP‐1), Scale bar: 50 µm; K) TNF‐α and L) IL‐6 in HUVECs by enzyme‐linked immunosorbent assay (a: PBS, b: LPS, c: LPS + Ir‐1@S‐R NPs, d: LPS + Ir‐4@S‐R NPs, e: LPS + PAG + Ir‐1@S‐R NPs, f: LPS + PAG + Ir‐4@S‐R NPs). Data are represented as mean ± SD (n = 3). The data were analyzed by unpaired 2‐tailed Student's *t*‐test using GraphPad Prism 7 (C, G, I, K and L). *p < 0.05, **p < 0.01, ***p < 0.001.

Excellent H_2_S release and ROS production ability is the foundation for in vitro thrombolysis. First, the blood compatibility of Ir‐1@S‐R NPs and Ir‐4@S‐R NPs was tested by hemolysis experiments (**Figures** **4**A and S29, Supporting Information). With the concentrations of Ir‐1@S‐R NPs and Ir‐4@S‐R NPs ranging from 0 to 200 µg mL^‒1^, all the blood samples showed relatively low hemolysis ratios of < 5%, indicating the good hemocompatibility of the NPs. To start the thrombolytic test, the targeting specificity of Ir‐4@S‐R NPs to thrombus was examined in vitro. Artificial blood clots were prepared by mixing fresh mouse blood with thrombus, then incubating with Ir‐4@S‐R NPs or non‐targeting Ir‐4@S NPs for 2, 4, or 6 h. After this time 200 µM ONOO^−^ was added, and the clots were visualized with an in vivo imaging system (IVIS). As expected, the blood clots incubated with Ir‐4@S‐R NPs exhibited remarkably stronger CL than Ir‐4@S NPs and PBS groups at each time point (Figure 4B,C) due the improved targeting mediated by the c(RGDfC) peptide component in the NPs. Then the thrombolytic effects were investigated by subjecting the blood clots to different treatments, that is, ultrasound, UK (urokinase), Ir‐1@S‐R NPs + ultrasound, and Ir‐4@S‐R NPs + ultrasound. In all the ultrasound‐treated groups, the blood clots were irradiated (1.0 W cm^−2^, 1 MHz, 50% duty cycle) for 40 min. After exposure to Ir‐4@S‐R NPs + ultrasound, the blood clots significantly shrank and the supernatant turned to a blood‐red color (Figure 4D,E). In contrast, the blood blots in the other groups showed only slight or moderate dissolution. Further, to explore the disruptive effect of ROS on the fibronectin skeleton, the amount of collapsed skeleton and fragments were distinguished after incubating fluorescein isothiocyanate (FITC)‐labeled fibrin with the NPs and ultrasound irradiation. A significant disruption of the fibrin clot network was evidenced by the presence of collapsed skeletal framework for Ir‐4@S‐R NPs + ultrasound. In contrast, ultrasound or free UK did not effectively disrupt the fibrin skeleton. This indicates that excellent ROS generation of Ir‐4@S‐R NPs, to disrupt the fibrin clot, originated from the combination of AIE performance and the multinuclear iridium centers (Figure 4F). The thrombolytic efficiency was quantitatively analyzed by measuring the percentage of thrombus mass loss after different treatments. The thrombolytic rates for ultrasound, UK, Ir‐1@S‐R NPs + ultrasound, and Ir‐4@S‐R NPs + ultrasound sequentially increased from 8.7%, 17.1%, 50.3% and 76.9% (Figure 4G). There was a significant increase in the absorbance at 450 and 540 nm of the supernatant of the Ir‐1@S‐R NPs + ultrasound, and Ir‐4@S‐R NPs + ultrasound groups, indicating the dissociation of fibrin and hemoglobin after thrombolysis (Figure S30, Supporting Information). Obviously, the Ir‐4@S‐R NPs + ultrasound group showed the strongest thrombolytic ability in vitro, and the effect was much better than that of the drug UK.

To evaluate the safety of the NPs for Human Umbilical Vein Endothelial Cells (HUVECs), Ir‐1@S‐R NPs or Ir‐4@S‐R NPs were co‐cultured with HUVECs for 24 h. Then the cells’ viability was analyzed using 3‐(4,5‐dimethylthiazol‐2‐yl)‐2,5‐diphenyltetrazolium bromide (MTT) assay, which showed that >90% of the cells remained alive after treating with 0–200 µg mL^‒1^ of either Ir‐1@S‐R NPs or Ir‐4@S‐R NPs demonstrating their negligible cytotoxicity (Figure 4H). Treatment of the HUVECs for 10 min with ultrasound intensities up to 1.0 W cm^−2^ hardly affected the cells’ viability, while 1.5 W cm^−2^ led to significant cell death (Figure 4I). Therefore, ultrasound intensity of 1.0 W cm^−2^ was used for further experiments.

The release of H_2_S from Ir‐4@S‐R NPs at the intracellular level was monitored with Washington State Probe‐1 (WSP‐1) reagent for PBS, US, Ir‐1@S‐R NPs and Ir‐4@S‐R NPs in HUVECs. As expected, bright green fluorescence was clearly observed only with Ir‐4@S‐R NPs (Figure 4J) where Ir‐4 acts as a bioorthogonal sonocatalyst to deliver H_2_S in situ. Inspired by the above results the anti‐inflammatory effect of Ir‐4@S‐R NPs was examined. 1 µg mL^−1^ of lipopolysaccharides (LPS) was used to induce the activation of RAW 264.7 cells. Significant reductions in interleukin‐6 (IL‐6) and tumor necrosis factor (TNF‐α) expression were observed after the treatment with Ir‐4@S‐R NPs. However, after 100 µM H_2_S‐specific inhibitor DL‐propargylglycine (PAG) was added, the inflammatory factors in the Ir‐4@S‐R NPs treatment group were not effectively down‐regulated, demonstrating that H_2_S generated through the bioorthogonal sonocatalytic reaction catalyzed by Ir‐4@S‐R NPs is an important factor in reducing cellular inflammation, not, for example, ROS or nanomaterials per se (Figure 4K,L). We conducted A) Gene ontology enrichment analysis, B) Reactome enrichment analysis and C) Kyoto encyclopedia of genes and genomes enrichment analysis of the LPS group versus the treatment group through transcriptomics as shown in Figure S31, Supporting Information. It was proved that the hydrogen sulfide gas in the treatment group successfully inhibited the activation of NF‐κB. The Western blot experiment shown in Figure S32, Supporting Information also demonstrated the anti‐inflammatory therapeutic effect of hydrogen sulfide gas in the treatment group. The above direct evidence provided for H_2_S‐mediated signaling clarifies the anti‐inflammatory pathway.

**Figure 5:**
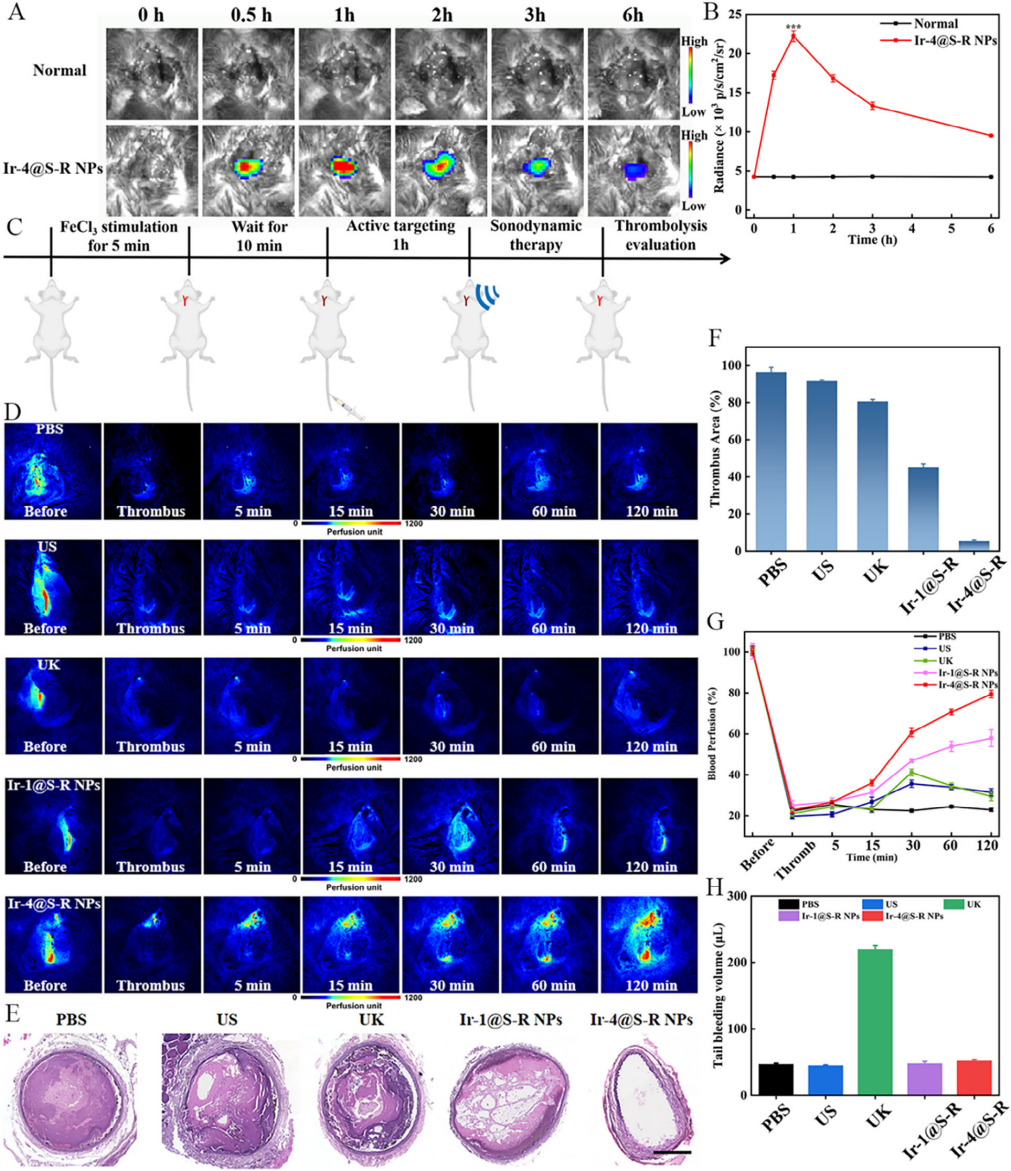
A) IVIS images of carotid artery site in thrombus mouse and normal mouse post‐injection of Ir‐4@S‐R NPs (5 mg kg^−1^); B) In vivo persistent intensity curves of carotid artery site in thrombus mouse and normal mouse post‐injection of Ir‐4@S‐R NPs (5 mg kg^−1^). Data are presented as mean ± SD (n = 3 independent experiments). *p < 0.05, **p < 0.01, and ***p < 0.001 versus control group; C) Experimental scheme of the thrombus model construction, antithrombusotic treatment, and thrombolytic effect evaluation; D) Representative LSBFMS analysis of the mouse carotid artery after FeCl3 induction and different therapeutic treatments with PBS, ultrasound, free UK, Ir‐1@S‐R NPs + ultrasound and Ir‐4@S‐R NPs + ultrasound; E) Hematoxylin‐eosin staining evaluated the thrombus resolution in the carotid artery following various treatments. Scale bar: 300 µm; F) Quantification of the calculated area of thrombus in the carotid artery after different treatments. Data are presented as mean ± SD (n = 3 independent experiments); G) The corresponding relative blood perfusion of the mouse carotid artery after FeCl_3_ induction and different therapeutic treatments with PBS, ultrasound, free UK, Ir‐1@S‐R NPs + ultrasound and Ir‐4@S‐R NPs + ultrasound. Data are presented as mean ± SD (n = 3 independent experiments); H) The mouse tail bleeding volume after different treatments. Data are represented as mean ± SD (n = 3). The data were analyzed by unpaired 2‐tailed Student's *t*‐test using GraphPad Prism 7 (B). *p < 0.05, **p < 0.01, ***p < 0.001.

Accurate thrombus‐targeting is crucial for the success of both SDT and anti‐inflammatory therapy. After intravenous injection of Ir‐4@S‐R NPs (5 mg kg^−1^) into mice, real‐time chemiluminescence images were captured by an IVIS system. Significant CL signals were observed at the thrombus site in the Ir‐4@S‐R NPs group, in contrast to normal vessels (**Figure** **5**A,B). The CL signal reached a maximum intensity after 1 h, indicating the long retention time of Ir‐4@S‐R NPs. We subsequently investigated Ir‐4@S‐R NPs for thrombus therapy. The in vivo effect of different formulations in mouse carotid artery thrombus models was evaluated according to the experimental scheme shown in Figure 5C. A Laser Speckle Blood Flow Monitoring System (LSBFMS) imaged and quantified the hemodynamic changes in the vasculature via live capture. First, the changes in blood after the induction of a carotid artery thrombus model were analyzed. After exposure of the carotid artery to 10% aqueous FeCl_3_ solution for 5 min, the blood flow in the model vessel was dramatically retarded, and a complete and stable vascular embolism was formed after 10 min (Figure 5D). We evaluated the influence of different doses of drugs on the effect of thrombolytic therapy. As shown in Figure S33, Supporting Information, an effective therapeutic effect cannot be achieved at a low dose of 1 mg kg^−1^. Although a dose of 3 mg kg^−1^ can restore blood flow, it still did not achieve the maximum blood volume recanalization. When the drug dose was 5 mg kg^−1^, effective thrombolysis and blood flow recovery were achieved within 2 h. Then different formulations, including PBS, Ir‐1@S‐R NPs (5 mg kg^−1^) and Ir‐4@S‐R NPs (5 mg kg^−1^), were separately administrated into the mouse via the tail vein. For comparation, free UK was also intravenously injected into the mouse at a dose of 0.04 mg kg^−1^. Free UK dissolved the clot and restored the blood flow to some extent, but the flow decreased after 30 min, as a clot again blocked the vessel due to the short life‐time of macromolecular protein drugs in blood. Figure 5G shows that ultrasound treatment had only a slight antithrombotic effect, and the blood vessels were rapidly blocked again after the treatment, indicating that ultrasound alone was not potent enough for thrombus eradication and suffered from a high risk of thrombus reformation. For the Ir‐1@S‐R NPs group, although more efficient thrombus removal was achieved, the blood perfusion was gradually restored to about 58% within 2 h and there was no reobstruction during the tested period. Notably, under ultrasound irradiation, Ir‐4@S‐R NPs, with both thrombus‐binding property and multimodal therapeutic effect, clearly showed the most efficient blood clot dissolution with the blood flow restored to about 77%. These results indicate that the synergism of SDT and H_2_S release significantly promotes thrombus therapy. In addition, infrared images also demonstrated that ultrasound does not cause thermal damage to the surrounding tissue of the mouse (Figure S34, Supporting Information). The mouse carotid arteries were collected for hematoxylin‐eosin (H&E) staining to assess the therapeutic efficacy by measuring the thrombus area (Figure 5E,F). The thrombolysis rates in the Ir‐1@S‐R NPs and Ir‐4@S‐R NPs groups were 56% and 91%, respectively. To assess whether the Ir‐4@S‐R NPs thrombolysis treatment had a risk of hemorrhagic side effects the mice were first intravenously injected with PBS, free UK, or different NPs, and then their tails were cut by a scalpel and the bleeding time was monitored. For the free UK group, the bleeding time significantly increased (about 20.9 min), which was 3.9‐fold longer than that of the PBS‐injected group (about 5.3 min). The NPs groups displayed a similar bleeding time to the PBS group (≈5.6−6.7 min) and a similar bleeding volume (Figure 5H, Figure S35, Supporting Information). These data prove that Ir‐4@S‐R NPs exhibited a good biosafety profile without the high bleeding risk of UK. Evans blue dye permeability experiments are shown in Figure S36, Supporting Information. The fluorescence intensity of the carotid artery of mice in the normal group was almost the same as the mice in the NPs‐treated group, which proved that the intensity of ultrasound (1.0 W cm^−^^2^) did not cause any significant thermal and mechanical damage to vascular endothelial cells.

**Figure 6:**
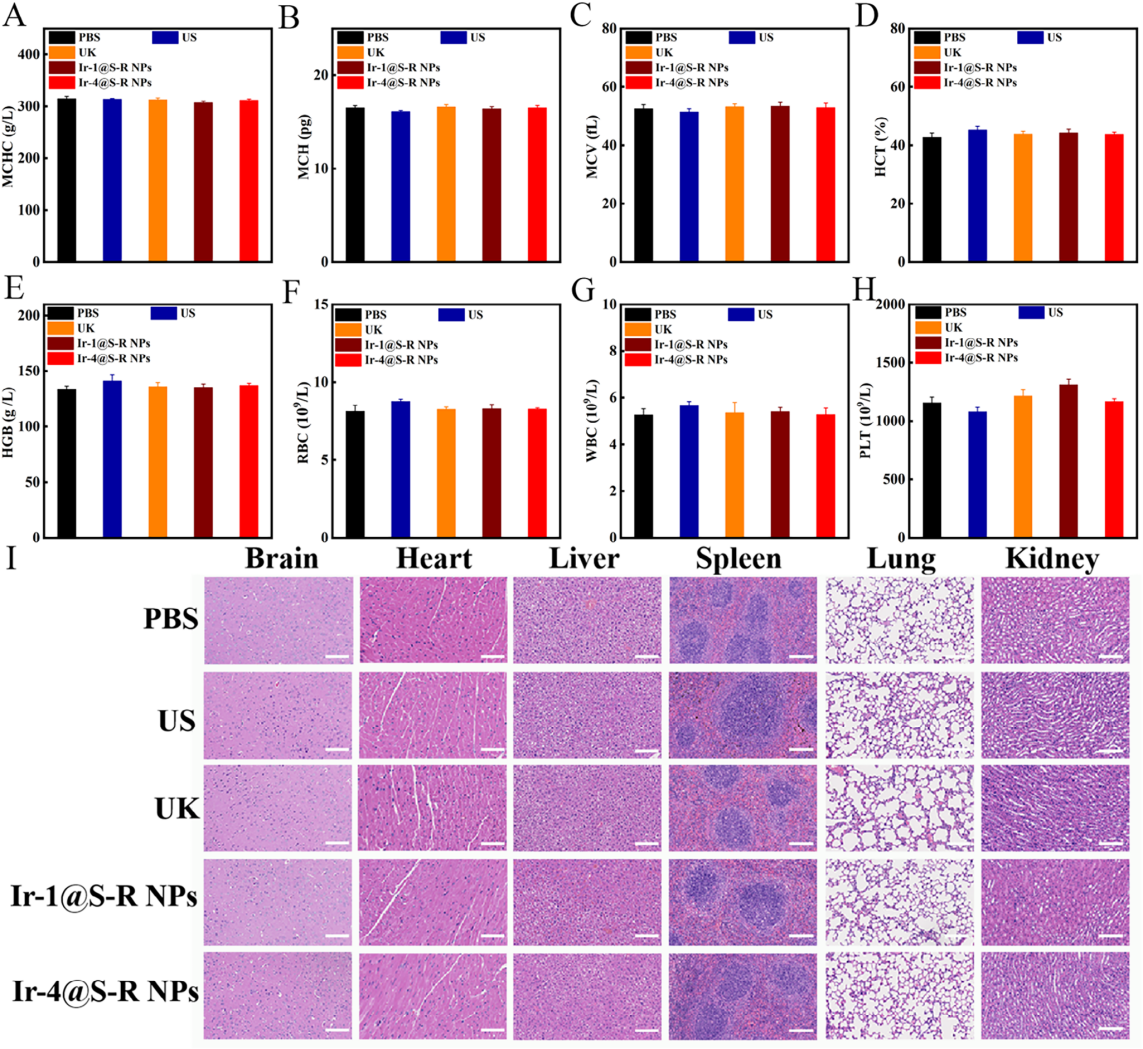
Blood test parameters regarding A) MCHC, B) MCH, C) MCV, D) HCT, E) HGB, F) RBC, G) WBC, H) PLT count of mice with different treatments after 3 days. Error bars, mean ± SD (n = 3). I) H&E staining of various organs from mice after different treatments. Representative H&E‐stained images from brain, heart, liver, spleen, lung and kidney slices. Scale bars = 100 µm. Data are represented as mean ± SD (n = 3).

Finally, blood tests showed that the hematological indexes of the mice treated with Ir‐4@S‐R NPs were within the normal range (**Figure** **6**A–H). Moreover, multiple serum biochemical markers for liver and renal damage, namely blood urea nitrogen (BUN), creatinine (CREA), alanine aminotransferase (ALT) and aspartate aminotransferase (AST) were measured as shown in Figure S37, Supporting Information. These hepatorenal indicators showed no significant differences between PBS‐ and NPs‐treated mice. Meanwhile, the biocompatibility of Ir‐4@S‐R NPs was evaluated. Ir‐4@S‐R NPs are mainly metabolized by the liver and kidney (Figure S38, Supporting Information). A metabolic kinetics distribution study via ICP‐MS revealed that the NPs mainly accumulated in liver, lung and kidney and the accumulated content in these organs decreased significantly by 7 days after injection (Figure S39, Supporting Information). The in vivo safety profile of Ir‐4@S‐R NPs was subsequently assessed after treatment with PBS, ultrasound, UK, Ir‐1@S‐R NPs or Ir‐4@S‐R NPs. The major organs, including brain, heart, liver, spleen, lung and kidney were collected for post‐mortem examination. The histological images of all the organs showed no detectable pathological changes or inflammatory reactions after treatment with Ir‐4@S‐R NPs (Figure 6I). All the experiments fully demonstrate that Ir‐4@S‐R NPs have good biosafety in vivo, which is an important prerequisite for their future clinical applications.

**Figure 7:**
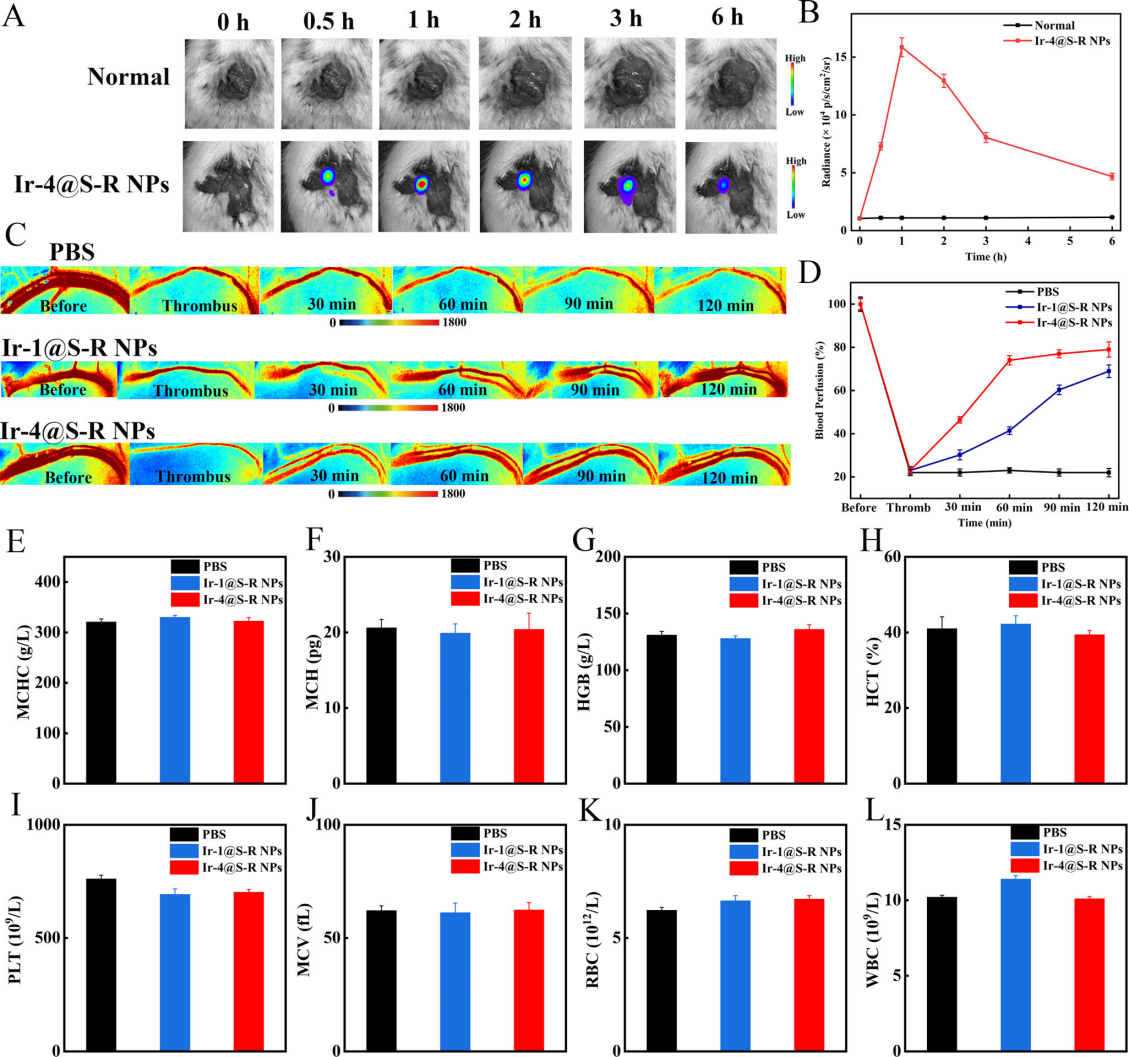
A) IVIS images of normal rat and femoral vein site thrombus rat post‐injection of Ir‐4@S‐R NPs (5 mg kg^−1^); B) In vivo persistent intensity curves of normal rat and femoral vein site thrombus rat post‐injection of Ir‐4@S‐R NPs (5 mg kg^−1^). Data are presented as mean ± SD (n = 3 independent experiments); C) Representative LSBFMS analysis of the rats femoral vein after FeCl_3_ induction and different therapeutic treatments with PBS, Ir‐1@S‐R NPs + ultrasound and Ir‐4@S‐R NPs + ultrasound; D) The corresponding relative blood perfusion of the rats femoral vein after FeCl_3_ induction and different therapeutic treatments with PBS, Ir‐1@S‐R NPs + ultrasound and Ir‐4@S‐R NPs + ultrasound. Data are presented as mean ± SD (n = 3 independent experiments); E) MCHC, F) MCH, G) HGB, H) HCT, I) PLT, J) MCV, K) RBC, L) WBC count of rats with different treatments after 3 days. Data are represented as mean ± SD (n = 3).

In addition to mouse carotid artery thrombosis, the lower extremity femoral vein thrombosis model of rats was established. First, we verified that the real‐time CL images were captured by an IVIS system after the injection of Ir‐4@S‐R NPs (5 mg kg^−1^) into rats. Significant CL signals were observed at the thrombus site in the Ir‐4@S‐R NPs group, in contrast to normal vessels (**Figure** **7**A,B). The CL signal also reached a maximum intensity after 1 h, indicating the long retention time of Ir‐4@S‐R NPs. These experiments further prove the in vivo potential of Ir‐4@S‐R NPs for deep thrombus imaging for clinical applications. We subsequently investigated Ir‐4@S‐R NPs for rats’ lower extremity femoral thrombus therapy. After exposure of the femoral vein to 10% aqueous FeCl_3_ solution, the blood flow in the model vessel was dramatically retarded, and a complete and stable vascular embolism was formed after 10 min (Figure 7C). Then the different treatment groups, including PBS, Ir‐1@S‐R NPs (5 mg kg^−1^) and Ir‐4@S‐R NPs (5 mg kg^−1^), were separately administrated into the rat via the tail vein. For the Ir‐1@S‐R NPs group, the blood perfusion was gradually restored to about 67% within 2 h and there was no reobstruction during the tested period. Notably, Ir‐4@S‐R NPs under ultrasound irradiation, with both thrombus‐binding property and multimodal therapeutic effect, clearly showed the most efficient blood clot dissolution, with the blood flow restored to about 79% (Figure 7D). These data indicate that Ir‐4@S‐R NPs have excellent thrombus removal capability for multiple thrombus models.

Meanwhile, complete blood panel tests were conducted. The hematology parameters of the rats that received NPs were all within the normal ranges (Figure 7E–L). Multiple serum biochemical markers for liver and renal damage, including blood urea nitrogen (BUN), creatinine (CREA), alanine aminotransferase (ALT) and aspartate aminotransferase (AST), were measured for analyzing the liver and kidney functions (Figure S40, Supporting Information). These hepatorenal indicators showed no significant differences between PBS‐ and NPs‐treated rats. Ir‐4@S‐R NPs are mainly metabolized by the liver and kidney (Figure S41, Supporting Information). A metabolic kinetics distribution study via ICP‐MS revealed that the NPs mainly accumulated in the liver, lung and kidney. The accumulated content in these organs decreased significantly by 7 days after injection (Figure S42, Supporting Information). All the experimental results fully demonstrate that Ir‐4@S‐R NPs have good biosafety in rats.

## 3 Conclusion

In summary, we present a novel nanoplatform that integrates NIR chemiluminescence imaging for thrombosis detection, sonodynamic thrombus therapy and on‐demand release of H_2_S. An AIE‐active Ir(III) complex‐based sonosensitizer with excellent ROS production and sonocatalytic ability is the basis of this breakthrough in thrombosis treatment. The nanoplatform emits NIR CL triggered by endogenous ONOO^−^ and possesses deep tissue penetration (12 mm) in chicken breast experiments in vitro. The combination of SDT and H_2_S release not only potently dissolved blood clots, but also promoted anti‐inflammation efficiency. Ir‐4@S‐R NPs achieved a thrombolytic rate of 76.9% in vitro. The NPs accomplished the rapid and efficient removal of blood clots in FeCl_3_‐induced mouse carotid thrombus models with almost complete recovery of blood flow, eliciting a significantly improved thrombolysis effect compared with free UK. Similarly, in a rat model of femoral vein thrombosis, the thrombus was almost completely ablated and blood flow recanalization was achieved. The nanoplatform has favorable biocompatibility in vivo and low risk of hemorrhagic side effects, representing a new and safer thrombolytic therapy. We envisage these results will inspire new strategies for diagnosing and treating life‐threatening diseases caused by various thrombusotic disorders.

## 4 Experimental Section

### Animal Experiments

All animal experiments were approved by the Ethics Committee for Animal Experimentation of China Technology Industry Holdings (Shenzhen) Co., Ltd (Ethics approval number: 20 240 030). All procedures complied with the Animal Research: Reporting In Vivo Experiments (ARRIVE) guidelines. The mice and rats were housed in a pathogen‐free environment with no more than three animals per cage. They were subjected to a 12‐h dark‐light cycle and were given unrestricted access to food and water.

### Statistical Analysis

Data are denoted as the mean ± standard deviation (SD). The significance between experimental and control groups was determined by unpaired 2‐tailed Student's *t*‐test using the GraphPad Prism 7 software. A value of *p* < 0.05 was considered statistically significant. * *p* < 0.05, ** *p* < 0.01,*** *p* < 0.001.

## Conflict of Interest

The authors declare no conflict of interest.

## Supporting information



Supporting Information

## Data Availability

The data that support the findings of this study are available in the supplementary material of this article.
