# Supplementary material for: Sonodynamic and Bioorthogonal Sonocatalytic Thrombotic Therapy Based on AIE Cationic Tetranuclear Ir(III) Complex Nanoplatform Guided by NIR‐Chemiluminescence Imaging
Source: Adv Mater. 2025 Jul 10;37(39):2503599. doi: 10.1002/adma.202503599 (PMC12506604; doi:10.1002/adma.202503599)
Supplement: Supplementary file 1 — Supporting Information [file ADMA-37-2503599-s001.pdf]

ADVANCED MATERIALS

Supporting Information

for *Adv. Mater.*, DOI 10.1002/adma.202503599

Sonodynamic and Bioorthogonal Sonocatalytic Thrombotic Therapy Based on AIE Cationic Tetranuclear Ir(III) Complex Nanoplatform Guided by NIR-Chemiluminescence Imaging

Zihan Wu, Liping Zhang, Ziwei Wang, Shengnan Liu, Qiaohua Zhang, Chunguang Shi, Yachao Wang, Gelin Xu, Dongxia Zhu*, Martin R. Bryce*, Lijie Ren* and Ben Zhong Tang**

Supporting Information

Sonodynamic and Bioorthogonal Sonocatalytic Thrombotic Therapy Based on AIE Cationic Tetranuclear Ir(III) Complex Nanoplatform Guided by NIR-Chemiluminescence Imaging

Zihan Wu, Liping Zhang, Ziwei Wang, Shengnan Liu, Qiaohua Zhang, Chunguang Shi, Dongxia Zhu*, Martin R. Bryce*, Lijie Ren* and Ben Zhong Tang**

General Materials and Methods.

All the chemicals were obtained from Sigma-Aldrich unless otherwise specified. The solvents for chemical reactions were distilled before use. Materials for organic synthesis were purchased from Energy Chemical Company. 1,2-Distearoyl-sn-glycero-3-phosphoethanolamine-*N*-[maleimide(poly(ethyleneglycol))-2000] (DSPE-PEG-MAL) was purchased from Laysan Bio, Inc. (Arab, AL). c(RGDfC) was supplied by Hangzhou Special Peptide Biotechnology Co. 1,3-Diphenylisobenzofuran (DPBF) was purchased from Energy Chemical Company. Urokinase (UK, 1.2 × 105 IU/mg) was obtained from Aladdin Biochemical Technology Company Ltd. (Shanghai, China). 3-(4,5-dimethyl-2-thiazolyl)-2,5-diphenyl-2*H*-tetrazolium bromide (MTT) was purchased from Shanghai Beyotime Biotechnology Co. Ltd. Enzyme-linked immunosorbent assay (ELISA) kit was provided by Biolegend.

Reactions were monitored with analytical thin-layer chromatography (TLC) on silica. ^{1}H NMR spectra were recorded at 25 °C on a Varian 500 MHz spectrometer. Mass spectra were recorded on a Bruker autoFlex III instrument. Elemental analysis was obtained on EA3000. UV-vis absorption spectra were recorded on a Shimadzu UV-3100 spectrophotometer. The photoluminescence spectra were recorded on an Edinburgh FLS920 spectrofluorimeter under air at room temperature. Transmission electron microscopy (TEM) images were taken by a TECNAI F20 microscope. Diameter and diameter distribution of the nanoparticles were determined by a Malvern Zetasizer Nano instrument for dynamic light scattering (DLS). Confocal laser scanning microscopy (CLSM) images were taken using a LSM 800 Zeiss, Germany. Chemiluminescence and fluorescence were acquired by an in vivo imaging system (Xenogen IVIS Lumina II system).

Preparation of NPs.

The nanoparticles were prepared by a modified nanoprecipitation method. The synthesis process was carried out at 25 °C. First, Ir-1 (1 mg), H_2S-N_3 (0.5 mg) and DSPE-PEG-MAL (3 mg) were dispersed in 1 mL of THF. After the compounds were completely dissolved, the above solution was added dropwise to 10 mL of purified

water under vigorous stirring. Stirring was continued overnight in a fume hood to evaporate the organic solvent. Then c(RGDfC) was dispersed in 1 mL of water and the peptide was loaded by thiol-Michael addition reaction for 16 h at room temperature. The residual c(RGDfC) peptide was then removed using a dialysis bag. The obtained Ir-1@S-R NPs were stored in a refrigerator at 4 °C for subsequent experiments. Ir-4@S-R NPs were prepared in the same way using Ir-4 complex.

Preparation of Reactive Oxygen and Nitrogen Species (RONS) Solutions.

The RONS solutions containing H_2O_2, TBHP and ClO^-, respectively, were purchased and diluted to the experimental concentration (200 μM) by 1×phosphate buffered saline (PBS) (pH 7.4). In brief, •OH was produced by addition of ferrous chloride (0.1 M, 1 mL) into H_2O_2 solution (1.0 M, 1 mL) through a Fenton reaction. Accordingly, the concentration of •OH is the same as that of Fe^{2+} (50 mM). $O_2^{\bullet-}$ was generated from KO_2 (35.5 mg), which was directly added into dimethyl sulfoxide (10 mL) at a final concentration of 50 mM. $ONOO^-$ was prepared by addition of sodium hydroxide (1.5 M) into mixtures of sodium nitrite (0.6 M), hydrogen peroxide (0.7 M) and hydrochloric acid (0.6 M) at 0 °C, followed by purification through a short column of manganese dioxide to remove excess hydrogen peroxide. The concentration of $ONOO^-$ was determined by measurement of the absorption at 302 nm: $c[ONOO^-] = Abs_{302\ nm}/1.67$ (mM). $^1O_2$ was produced by addition of ClO^- solution (100 mM, 1 mL) into H_2O_2 solution (200 mM, 1 mL). According to this reaction, the concentration of $^1O_2$ is the same as that of ClO^- (50 mM). NO_2^- was generated from $NaNO_2$ (35 mg), which was directly added into deionized water (10 mL) at a final concentration of 50 mM.

Chemiluminescence and Fluorescence Imaging In Vitro.

For in vitro chemiluminescence imaging, including determinations of RONS selectivity, chemiluminescence spectra, and $ONOO^-$ activated sensitivity, etc., the concentration of Ir-4@S-R NPs was 100 μg/mL. The chemiluminescence imaging was carried out with an Xenogen IVIS Lumina II system in a bioluminescent mode (exposure time for 60 s) post RONS (200 μM) addition, unless otherwise specified. Tests were every 20 nm/step by the different filters. The environmental temperature for in vitro afterglow imaging was kept at 37 °C. The fluorescence spectra of Ir-4@S-R NPs were measured by the same IVIS instrument in a fluorescent mode with excitation at 465 ± 10 nm (exposure time for 1 s). The fluorescence and chemiluminescence images were analyzed by region of interest (ROI) analysis using the Living Image 4.2 Software.

FITC-Labeled Fibrin Clots Assay.

Fluorescein isothiocyanate (FITC)-labeled fibrin clots were induced by the addition of 10 U mL^{-1} thrombin and 2.5 mM $CaCl_2$ into a fibrinogen solution containing 1 mg mL^{-1} fibrinogen (200 μL) and 1 mg mL^{-1} FITC-labeled fibrinogen (20 μL), followed by incubation at 37 °C for 1 h. The clot was incubated with Ir-4@S-R NPs (100 μg mL^{-1}, 600 μL) for 1 h and further irradiated with ultrasound (1.0 W cm^{-2}, 1 MHz, 50%

duty cycle) and evaluated through a CLSM.

In vitro Hemolysis Experiments.

A hemolysis assay was conducted to evaluate the hemocompatibility of Ir-4@S-R NPs. Firstly, the whole blood of a mouse treated with citrate was gathered and centrifuged at 3000 rpm for 3 min to obtain erythrocytes. Then, the erythrocytes were resuspended in an equal volume of PBS. PBS or water were used as negative and positive controls, respectively, which were added to 2 mL microtubes containing erythrocytes. Various concentrations (5, 10, 50, 100, 150 and 200 μg mL^{-1}) of Ir-4@S-R NPs were incorporated into the erythrocytes for comparison with the controls. The mixture was then incubated in a water bath at 37 ºC for 2 h, followed by centrifugation at 3000 rpm for 10 min. After centrifugation, 100 μL of supernatant from each sample was transferred to a 96-well plate and the absorbance was recorded with an enzyme marker at 540 nm. The equation for calculating the hemolysis rate (%) is presented below. Hemolysis rate (%) = $(A_0 - A_1) / (A_2 - A_1) \times 100\%$.

In vitro Thrombus Targeting of Ir-4@S-R NPs.

Fresh mouse blood was collected and divided into tubes with equal volumes (5 mL). Each tube was mixed with thrombin (5 U μL^{-1}) and $CaCl_2$ (3 mM) to induce the formation of clots and cut into equal sizes. The artificial thrombus was then incubated with PBS, Ir-4@S NPs (no c(RGDfC)) or Ir-4@S-R NPs aqueous solution (0.1 mg mL^{-1}) to verify the targeting ability of the NPs. After incubation for 2 h, 4 h or 6 h, the thrombus clots were taken out and washed three times with PBS. The chemiluminescence of the clots was analyzed using an IVIS imaging system.

In vitro Thrombolytic Efficacy.

The artificial thrombus was placed into a 5 mL glass vial, to which the mixture of 2.5 mL of PBS and 0.5 mL of the different NPs aqueous solutions was added. The mixture was irradiated with ultrasound (1.0 W cm^{-2}, 1MHz, 50% duty cycle). The weights of the thrombus before and after thrombolytic treatment were measured to calculate the thrombolysis rate, thrombolysis rate = (weight before treatment – weight after treatment)/weight before treatment.

In Vitro Cell Cytotoxicity Test.

The human umbilical vein endothelial cells (HUVECs) were obtained from Peking Union Medical College Hospital (Peking, China). Fetal bovine serum (FBS), Ham's F12K, heparin, endothelial cell growth supplement (ECGS), L-glutamine, penicillin, and streptomycin were obtained from Corning (New York, USA). The cells were maintained in Ham's F12K medium with heparin (0.1 mg mL^{-1}), ECGS (0.05 mg mL^{-1}), 10% FBS, 1% L-glutamine and 1% penicillin/streptomycin at 37 °C containing 5% CO_2. For in vitro cytotoxicity tests, HUVECs were seeded into 96 well plates with a density of 1×10^4 per well. After 24 h, the cells were incubated with NPs for another 24 h. The MTT assay was conducted following the standard protocol.

In vitro H_2S Release.
HUVEC were seeded in 96-well plates and allowed to adhere and proliferate for a period of 24 h. Following this incubation, the cells were treated with Ir-4@S-R NPs. After treatment, the intracellular H_2S content was assessed using the specific H_2S probe, WSP-1, which selectively binds to H_2S and facilitates its detection.

Enzyme-Linked Immunosorbent Assay.
The supernatants were collected from the different treatment groups of RAW 264.7 cells, and the concentrations of the inflammatory factors, namely brain necrosis factor TNF-α and interleukin IL-6 were measured using an enzyme-linked immunosorbent assay (ELISA) kit. Each experiment was performed independently three times.

Construction of mice carotid artery thrombus model.
Male C57 BL/6J mice (8-10 weeks old) were anesthetized using isoflurane (2% of isoflurane, 0.5 mL min^{-1} oxygen) and the neck hair was shaved off. Then the skin around the neck was cut with surgical scissors and the connective tissue and fat were peeled away to expose the carotid vessels. A 10% aqueous solution of $FeCl_3$-soaked filter paper (2 × 1 mm) was placed on the surface of the exposed carotid artery for 10 min, then the filter paper was removed. The vessel and surrounding tissue were washed with sterilized saline solution. An apparent aggregated embolus could be observed under a somatic microscope. A laser speckle imaging system (RWD, RFLSI III, Shenzhen, China) was used to monitor the hemodynamic changes before and after the induction of carotid artery thrombus using $FeCl_3$. All animal procedures were approved by the China Technology Industry Holdings (Shenzhen) Co., Ltd.

Construction of rats' femoral vein thrombus model.
Female SD rats (about 200 g) were anesthetized using isoflurane (2% of isoflurane, 0.5 mL min^{-1} oxygen). Then the skin around the thigh was cut with surgical scissors and peeled away to expose the femoral vein vessels. A 10% aqueous solution of $FeCl_3$-soaked filter paper (2 × 1 mm) was placed on the surface of the exposed femoral vein for 10 min, then the filter paper was removed. The vessel and surrounding tissue were washed with sterilized saline solution. An apparent aggregated embolus could be observed under a somatic microscope. A laser speckle imaging system (RWD, RFLSI III, Shenzhen, China) was used to monitor the hemodynamic changes before and after the induction of femoral vein thrombus using $FeCl_3$. All animal procedures were approved by the China Technology Industry Holdings (Shenzhen) Co., Ltd.

In vivo chemiluminescence imaging of thrombus.
The C57 BL/6J mice (8-10 weeks old) with a thrombus model or normal C57 BL/6J mice (8-10 weeks old) were intravenously administrated with Ir-4@S-R NPs (5 mg/kg). The mice were then anesthetized using isoflurane (2% of isoflurane, 0.5 mL min^{-1} oxygen), and placed on a heating platform at 37 °C. At predetermined times (0, 30, 60, 120, 180 and 360 min) post NPs administration, in vivo chemiluminescence

imaging was carried out using an IVIS imaging system.

Evaluation of the thrombolytic effect of different formulations in vivo.
To evaluate the therapeutic effect of different formulations on the thrombi, the mice with carotid thrombosis or lower extremity arterial thrombosis were randomly divided into five groups (n = 3): PBS, US, free UK, Ir-1@S-R NPs or Ir-4@S-R NPs. The different formulations were intravenously injected into the mouse via the tail vein. For the US-treated groups, the carotid arteries were treated with ultrasound irradiation (1.0 W cm^{-2}, 1MHz, 50% duty cycle) for 120 min at 60 min post NPs administration. At different times (0, 5, 15, 30, 60 and 120 min) after the treatments, a laser speckle imaging system was used to observe and record the blood flow velocity and blood flow at the thrombosis sites, and applied to measure the blood flow velocities. Finally, the mouse was euthanized, and the carotid arteries was excised for section and H&E staining. The areas and morphology of the thrombi after different treatments were observed under an optical microscope. The sections were analyzed using the Image J2x software (version 2.1.4.7) and thrombolytic efficiency was determined by the area ratio of vascular occlusion to total vascular lumen.

In vivo biosafety evaluation.
To test the potential toxicity of Ir-4@S-R NPs, healthy male C57 BL/6J mice (8-10 weeks old) were intravenously injected with PBS, free UK, Ir-1@S-R NPs or Ir-4@S-R NPs (200 μg mL^{-1}) or treated with US only. One week after PBS or NPs administration, the mice were euthanized and blood was withdrawn. Then the hematological parameters were analyzed using an automated hematology analyzer. The major organs (brain, heart, liver, spleen, lung, and kidneys) were also harvested for histological analysis. The tissues were fixed in 4% of paraformaldehyde (PFA), followed by embedding in paraffin and sectioning at a thickness of 5 μm for H&E staining. The slices were observed under a digital microscope (Leica QWin).

Synthesis.
Ligand L1 (Scheme S1): TPP-NH_2 (0.189 g, 0.3 mmol) and 2-pyridinecarboxaldehyde (0.039 g, 0.36 mmol) were added into ethanol (60 mL), and the mixture was stirred at reflux for 8 h. After cooling to room temperature, the precipitate was filtered and recrystallized with ethanol to obtain the target compound L1 with a yield of 83%.[1] ^{1}H NMR (500 MHz, $CDCl_3$, δ): 8.97 (s, 1H), 8.92 (d, J = 4.2 Hz, 2H), 8.88-8.81 (m, 7H), 8.40 (d, J = 7.7 Hz, 1H), 8.28 (d, J = 8.1 Hz, 2H), 8.24-8.20 (m, 6H), 7.94-7.91 (m, 1H), 7.80-7.74 (m, 9H), 7.70 (d, J = 7.8 Hz, 2H), 7.47-7.45 (m, 1H), -2.76 (s, 2H).

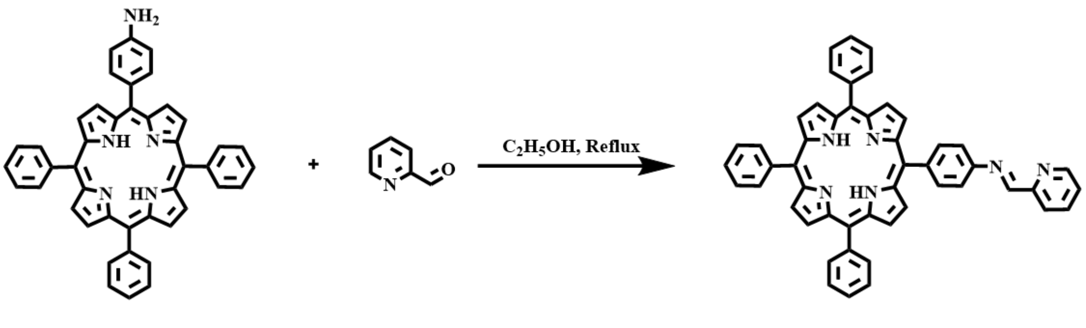

TPP-NH$_2$ **L1**

Scheme S1. Synthetic route to L1.

Ligand L4 (Scheme S2): L4 was synthesized similarly to L1, except that TPP-NH_2 was replaced by TPP-$(NH_2)_4$ with a yield of 78%.[1] ^{1}H NMR (500 MHz, $CDCl_3$, δ): 8.98-8.91 (m, 6H), 8.82 (d, J = 4.2 Hz, 2H), 8.41 (d, J = 6.0 Hz, 2H), 8.31-8.27 (m, 4H), 7.94-7.91 (m, 2H), 7.73-7.69 (m, 4H), 7.47-7.45 (m, 2H), -2.69 (s, 1H).

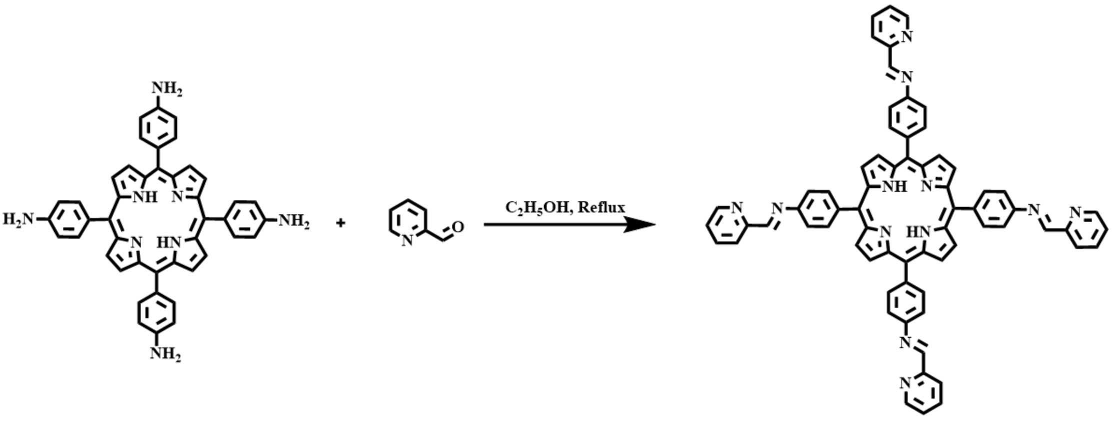

TPP(NH$_2$)$_4$ **L4**

Scheme S2. Synthetic route to L4.

Complex Ir-1 (Scheme S3): $[Ir(dF(CF_3)ppy)_2Cl]_2$[2] (0.149 g, 0.1 mmol) and auxiliary ligand L1 (0.144 g, 0.2 mmol) were dissolved in a mixture of methanol (30 mL) and dichloromethane (30 mL), and refluxed for 6 h under the protection of N_2. After cooling to room temperature, excess KPF_6 was added to the mixture (to replace counterion Cl^-). After stirring for 0.5 h, filtering to remove the excess potassium salts, and removal of the solvent from the filtrate by rotary evaporation, the crude product was obtained. Finally, the purple solid product was purified by column chromatography on silica gel (eluents dichloromethane /ethyl acetate 10/1, v/v) to give Ir-1 with a yield of 80%. ^{1}H NMR (500 MHz, DMSO-d_6, δ): 9.93 (s, 1H), 9.06 (s,1H), 8.86 (d, *J* = 26.8 Hz, 6H), 8.69 (d, J = 5.0 Hz, 1H), 8.58 (s, 2H), 8.52 (dd, *J* = 10.2, 3.3 Hz, 4H), 8.48 (d, *J* = 5.9 Hz, 1H), 8.23 (dd, *J* = 8.6, 4.2 Hz, 6H), 8.12 (d, *J* = 3.5 Hz, 1H), 8.02 (d, *J* = 5.5 Hz, 2H), 7.89 (d, *J* = 3.9 Hz, 1H), 7.87 (d, *J* = 5.5 Hz, 3H), 7.84 (dd, *J* = 8.4, 3.4 Hz, 5H), 7.80 (s, 1H), 7.22 (d, *J* = 5.5 Hz, 2H), 7.18 – 7.09 (m, 3H), 5.95 (dd, *J* = 5.4, 1.5 Hz, 1H), 5.87 (dd, *J* = 5.5, 1.5 Hz, 1H), -2.97 (s, 2H).

HRMS (ESI) *m/z*: $[M + H]^+$ calcd for $C_{74}H_{44}F_{10}IrN_8$,1427.32; found, 1427.23. Anal. Calcd. for $C_{74}H_{44}F_{16}IrN_8P$: C 57.21, H 3.24, N 6.93. Found C 57.19, H 3.23, N 6.93.

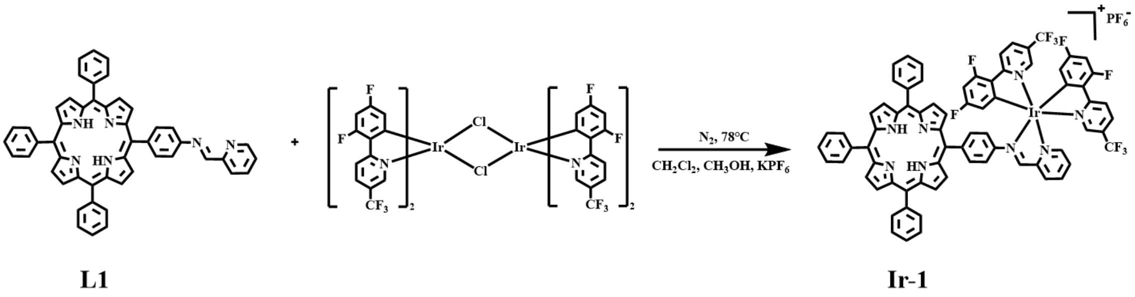

Scheme S3. Synthetic route to Ir-1.

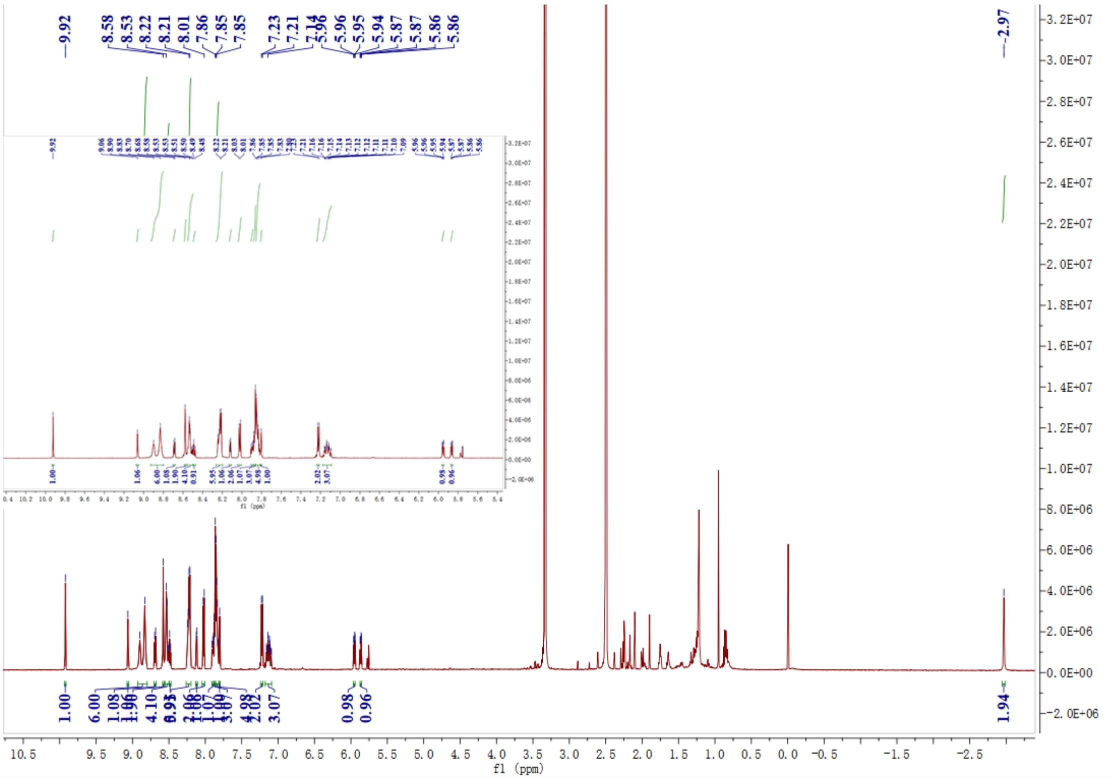

Figure S1. ^{1}H NMR spectrum of Ir-1 in DMSO-d_6 at room temperature.

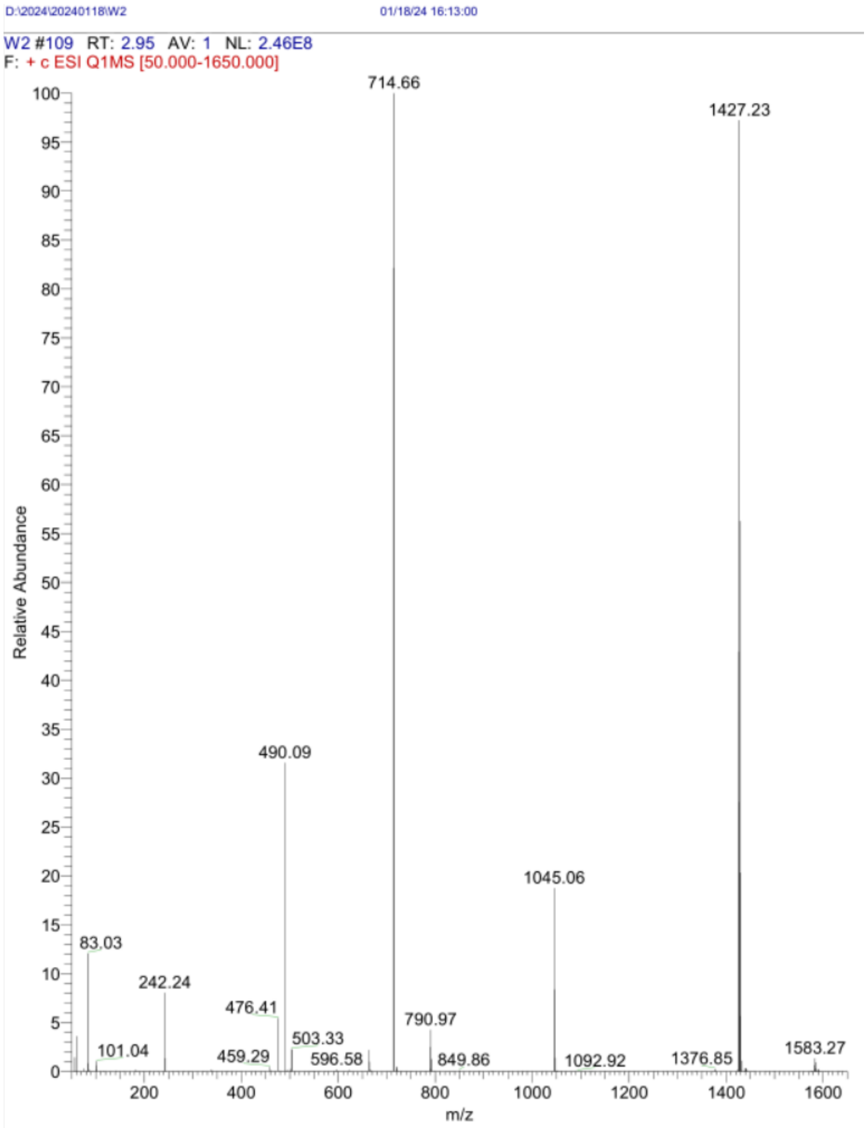

Figure S3. ESI mass spectrum of Ir-1 at room temperature.

Complex Ir-4 (Scheme S4): The synthesis and purification of Ir-4 was similar to that of Ir-1, $[Ir(dF(CF_3)ppy)_2Cl]_2$ (0.298 g, 0.2 mmol) and auxiliary ligand L4 (0.103 g, 0.1 mmol) were dissolved in a mixture of methanol (30 mL) and dichloromethane (30 mL) and refluxed for 8 h under the protection of N_2. After cooling to room temperature, excess KPF_6 was added to the mixture (to replace counterion Cl^-). After stirring for 0.5 h, filtering to remove the excess potassium salts, and removal of the solvent from the filtrate by rotary evaporation, the product Ir-4 was obtained pure without any further process, with a yield of 70%. ^{1}H NMR (500 MHz, DMSO-d_6, δ): 9.86 (s, 4H), 8.97 (s, 4H), 8.66 (d, J = 7.5 Hz, 4H), 8.53 (s, 20H), 8.45 (d, J = 6.4 Hz, 8H), 8.07 (d, J = 5.1 Hz, 4H), 8.00 – 7.93 (m, 8H), 7.89 – 7.84 (m, 4H), 7.72 (s, 4H), 7.21 (s, 8H), 7.10 (d, J = 10.0 Hz, 4H), 6.87 (s, 4H), 5.85 (d, J = 7.6 Hz, 4H), 5.75 (d, J = 7.8 Hz, 4H), -3.15 (s, 2H). HRMS (ESI) *m*/*z*: $[M + H]^{4+}$ calcd for $C_{164}H_{86}F_{40}Ir_4N_{20}$, 966.13; found, 966.3597. Anal. Calcd. for $C_{164}H_{86}F_{64}Ir_4N_{20}P_4$: C 50.96, H 2.24, N 7.25. Found C 50.95, H 2.23, N 7.24.

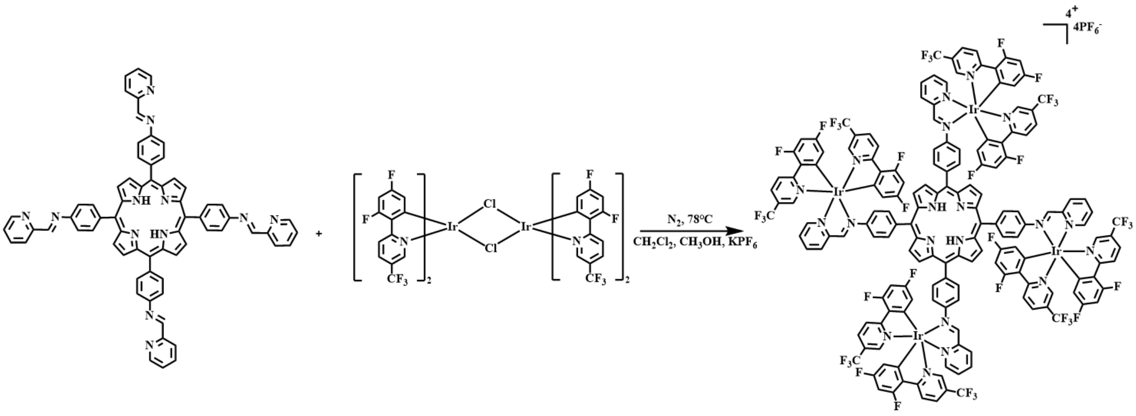

L4 **Ir-4**

Scheme S4. Synthetic route to Ir-4.

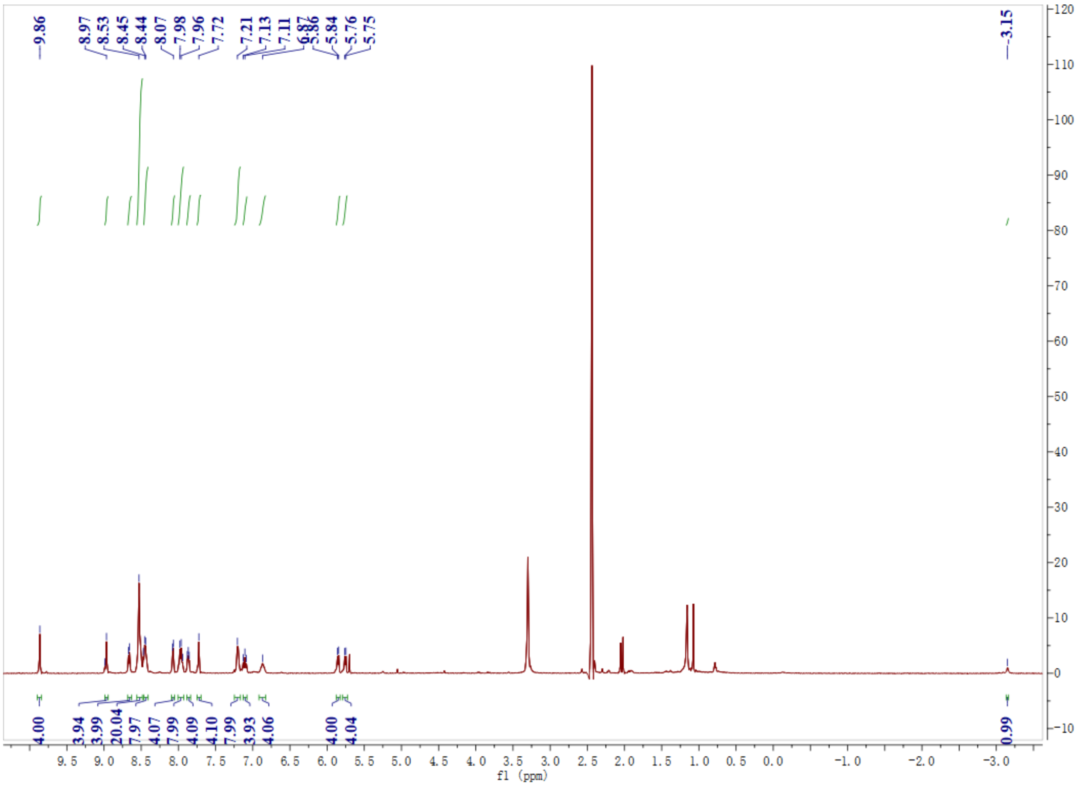

Figure S4. ^{1}H NMR spectrum of Ir-4 in DMSO-d_6 at room temperature.

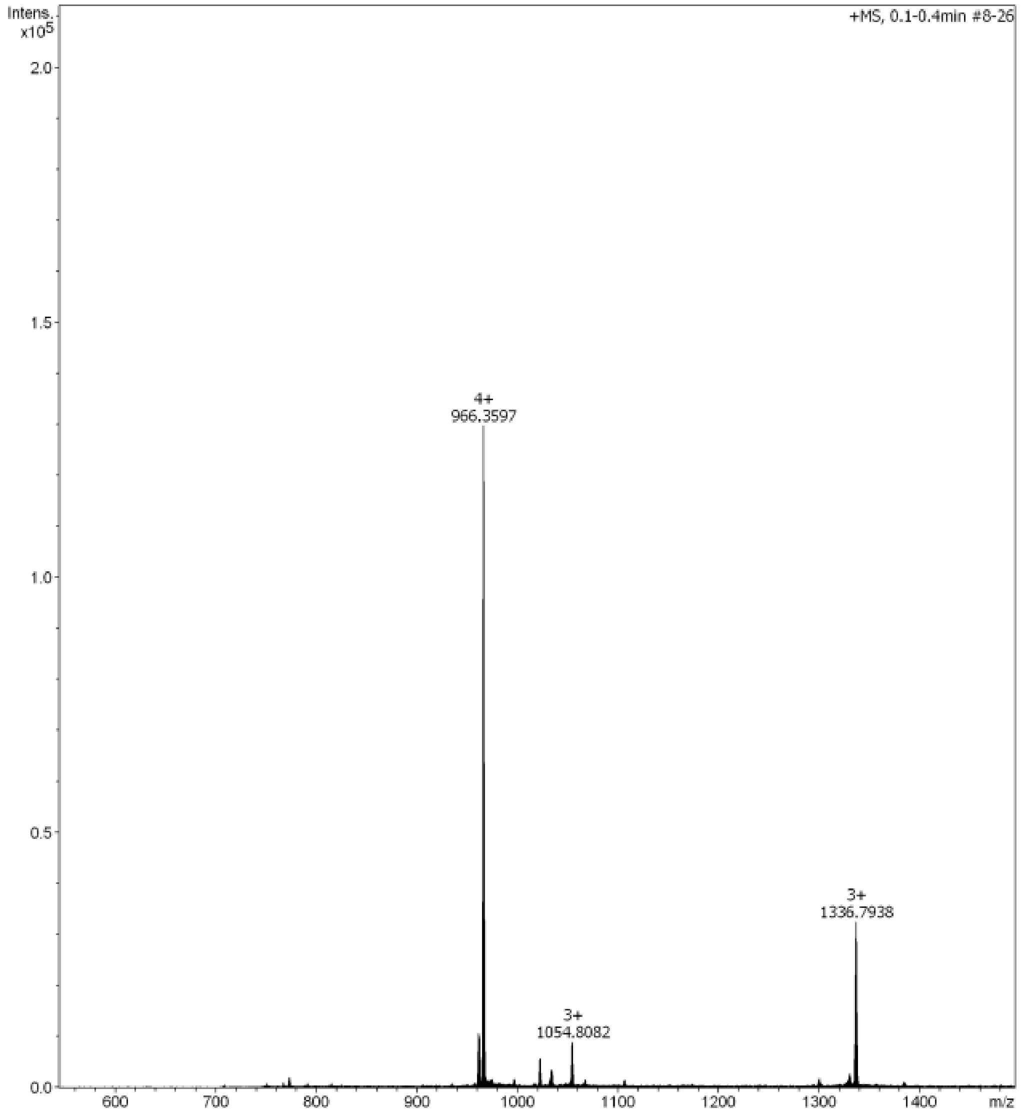

Figure S6. ESI mass spectrum of Ir-4 at room temperature.

Bisazide 1 (Scheme S5)[3]: Rhodamine 110 hydrochloride (“Rhodamine Chloride”, Sigma Aldrich; 50 mg, 0.136 mmol) was dissolved in water (4 mL) and HCl (12 N, 1 mL) under nitrogen atmosphere. Solid sodium nitrite (38 mg, 0.551 mmol) was added and the reaction mixture was stirred at ambient temperature. After 2 hours, NaN_3 (53 mg, 0.815 mmol) was added slowly to the above reaction mixture and the color of the solution changed from pink to yellow. The reaction was stirred at ambient temperature for a further 2.5 hours. Thereafter, the product was extracted into CH_2Cl_2 (3 x 20 mL), washed with brine, dried over Na_2SO_4, and concentrated in vacuo. The crude material was subjected to flash column chromatography with hexane/ethyl acetate (3:1) as the eluting solvent. The resulting diazide product 1 was isolated as a pale yellow solid (48.3 mg, 93%).^{1}H NMR (500 MHz, $CDCl_3$, δ): 8.04 (d, J = 7.5 Hz, 1H), 7.67 (dt, J = 25.1, 7.4 Hz, 2H), 7.14 (d, J = 7.5 Hz, 1H), 6.95 (s, 2H), 6.79 (d, J = 8.5 Hz, 2H), 6.73 (d, J = 8.5 Hz, 2H).

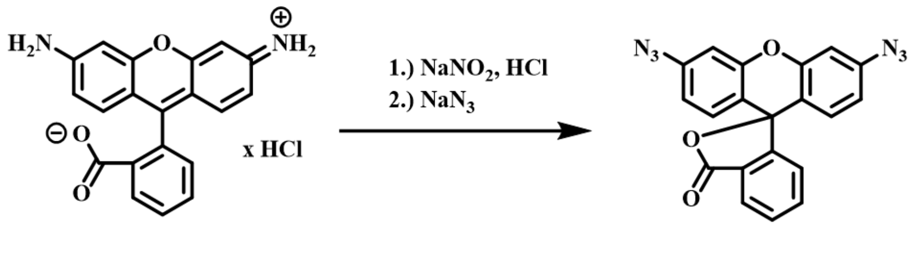

Rhodamine 110 hydrochloride **1**

Scheme S5. Synthetic route to Rhodamine bisazide 1**.**

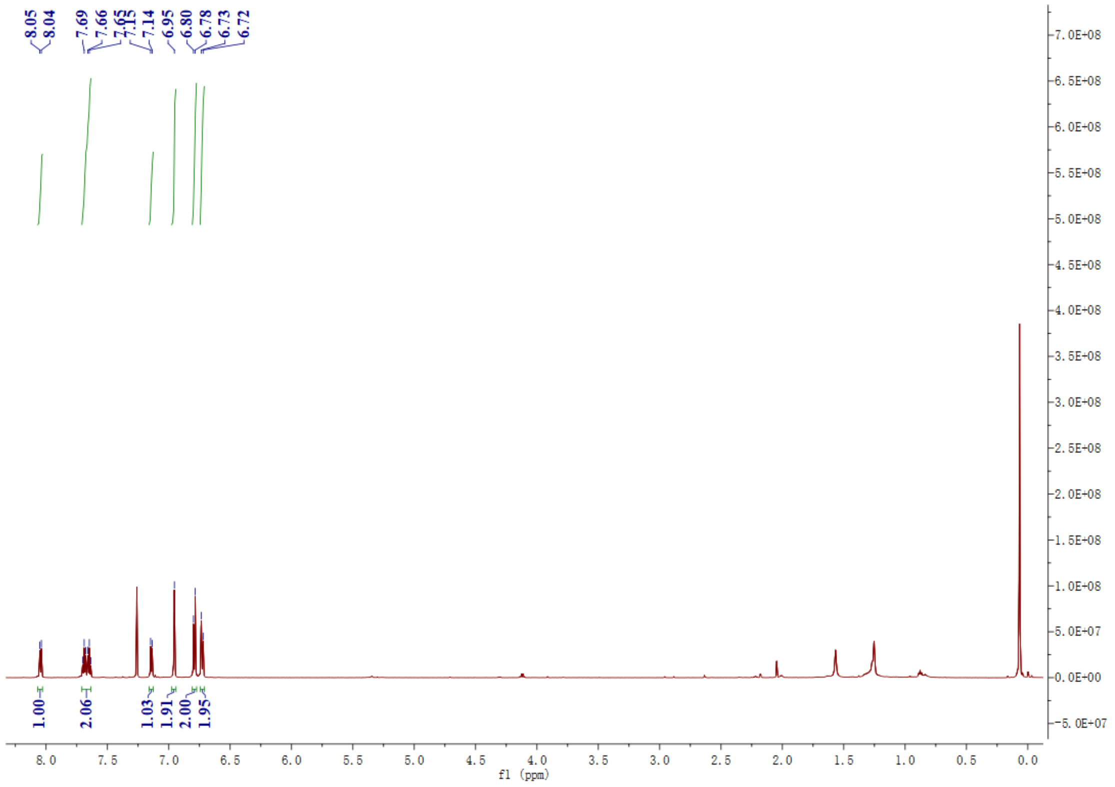

Figure S7. ^{1}H NMR spectrum of Rhodamine bisazide 1 in DMSO-d_6 at room temperature.

H_2S-N_3 (Scheme S6)[4]: To a solution of 4-azidobenzyl alcohol (5 mmol) and 4-fluorophenylisothiocyanate (5 mmol) in anhydrous tetrahydrofuran (25 mL) was added sodium hydride (60% in oil; 0.25 g, 6.25 mmol). The reaction mixture was stirred at room temperature in an atmosphere of nitrogen for 16 h. Then the tetrahydrofuran was removed under reduced pressure and the resulting material was partitioned between dichloromethane (30 mL) and water (30 mL). The organic layer was separated and dried over anhydrous magnesium sulfate. After evaporation of the solvent the residue was purified by silica gel column chromatography using petroleum/dichloromethane ether (10:7) as eluent. The resulting product H_2S-N_3 was isolated as a white solid. ^{1}H NMR (500 MHz, DMSO-d_6, δ): 11.19 (s, 1H), 7.61 (s, 1H), 7.45 (dd, J = 40.9, 6.8 Hz, 2H), 7.28 (s, 1H), 7.18 – 7.07 (m, 4H), 5.47 (d, J = 48.1 Hz, 2H).

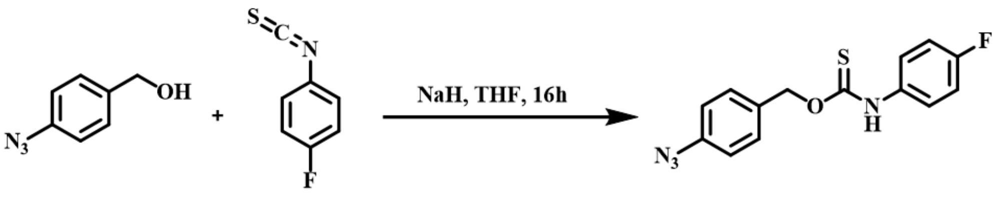

$H_2S\text{-}N_3$

Scheme S6. Synthetic route to $H_2S\text{-}N_3$.

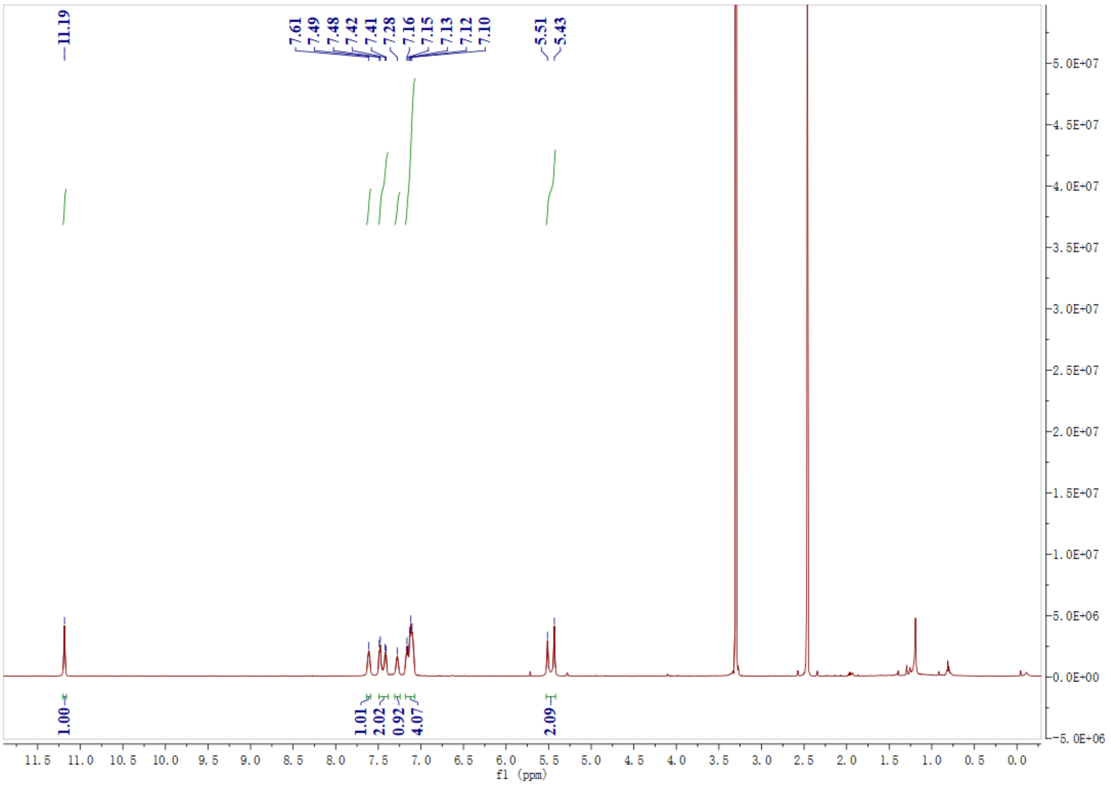

Figure S8. ^{1}H NMR spectrum of $H_2S\text{-}N_3$ in DMSO-d_6 at room temperature.

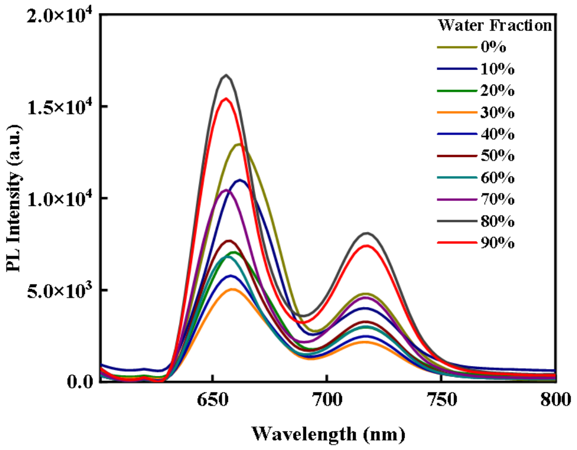

Figure S9. Emission spectra of Ir-1 in THF-H_2O mixtures (complex concentration = 1.0×10^{-5} M) with different water fractions (0%–90% v/v) at room temperature.

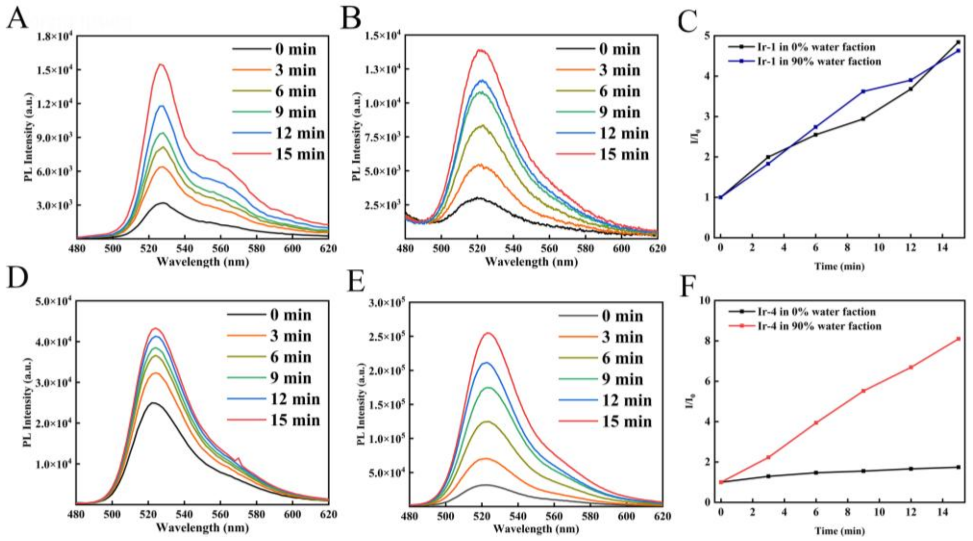

Figure S10. A) PL spectra changes of DCFH in the presence of Ir-1 (fw: 0%) upon ultrasound irradiation (1.0 W cm^{-2}, 1MHz, 50% duty cycle); B) in the presence of Ir-1 (fw: 90%) upon ultrasound irradiation (1.0 W cm^{-2}, 1MHz, 50% duty cycle); C) The change of PL intensity of DCFH with the change of time under different conditions in the presence of Ir-1. I_0 = initial intensity of 525 nm. I = real-time intensity of 525 nm; D) in the presence of Ir-4 (fw: 0%) upon ultrasound irradiation (1.0 W cm^{-2}, 1MHz, 50% duty cycle); E) in the presence of Ir-4 (fw: 90%) upon ultrasound irradiation (1.0 W cm^{-2}, 1MHz, 50% duty cycle); F) The change of PL intensity of DCFH with the change of time under different conditions in the presence of Ir-4. I_0 = initial intensity of 525 nm. I = real-time intensity of 525 nm.

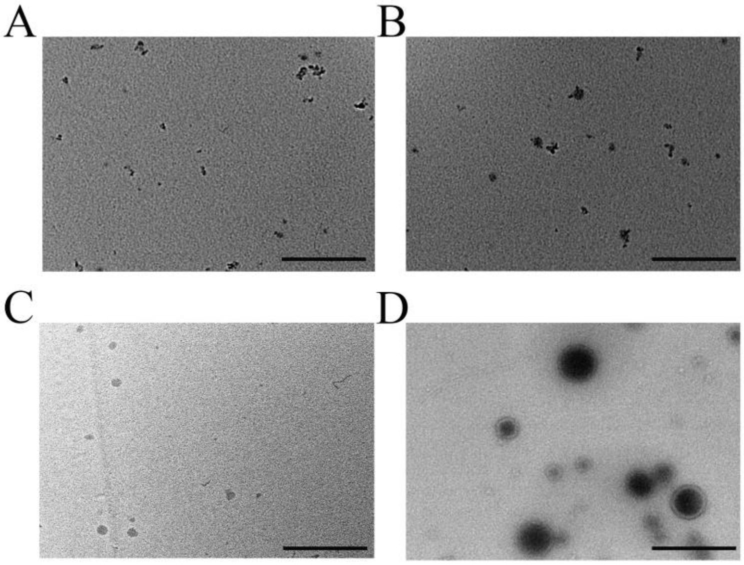

Figure S11. TEM images of A) Ir-1 when the water fraction is 0%; B) Ir-1 when the water fraction is 90%; C) Ir-4 when the water fraction is 0%; D) Ir-4 when the water fraction is 90%. Scale bar: 200 μm.

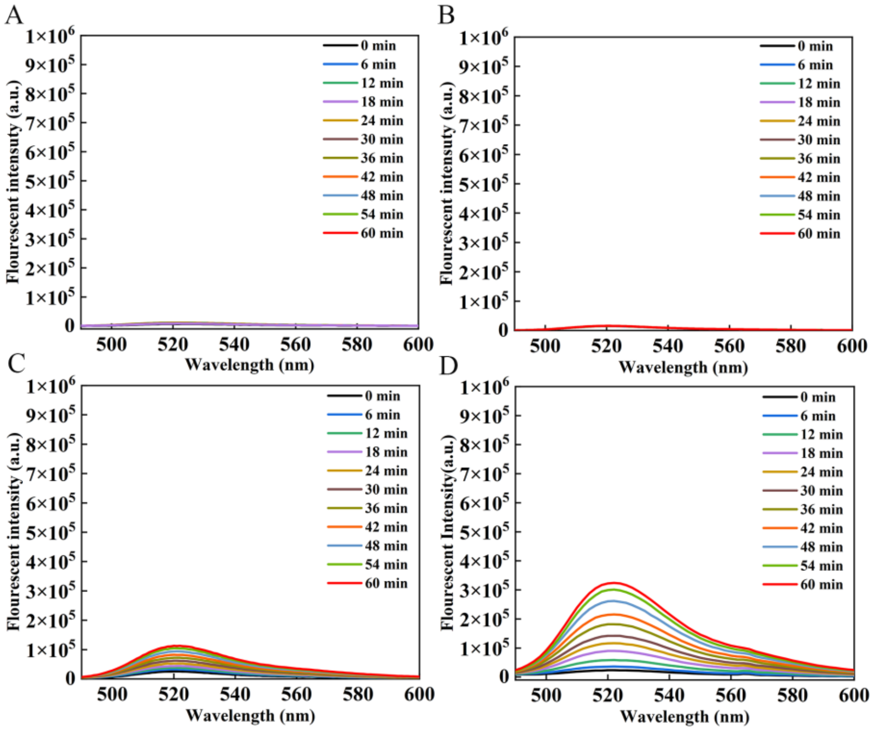

Figure S12. A) Fluorescence intensity of the reaction mixture of (Ir-1 = 5 μM, NaAsc = 2 mM, ultrasound irradiation (1.0 W cm^{-2}, 1MHz, 50% duty cycle)) in THF/H_2O at different time intervals without NaAsc; B) Fluorescence intensity of the reaction mixture of (Ir-1 = 5 μM, Rhodamine bisazide = 40 μM, ultrasound irradiation (1.0 W cm^{-2}, 1MHz, 50% duty cycle)) in THF/H_2O at different time intervals without ultrasound irradiation; C) Fluorescence intensity of the reaction mixture of (Ir-1 = 5 μM, Rhodamine bisazide = 40 μM, NaAsc = 2 mM) in THF/H_2O at different time intervals without Ir-4; D) Fluorescence intensity of the reaction mixture of (Ir-1 = 5 μM, Rhodamine bisazide = 40 μM, NaAsc = 2 mM, ultrasound irradiation (1.0 W cm^{-2}, 1MHz, 50% duty cycle)) in THF/H_2O at different time intervals.

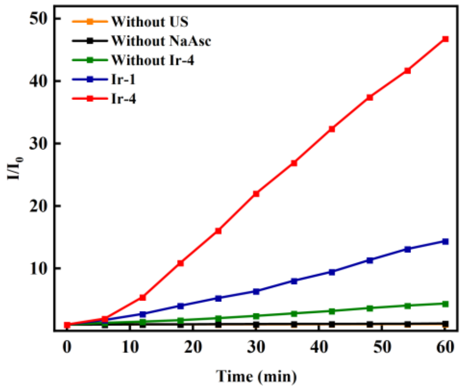

Figure S13. The change of PL intensity with the change of time of (Ir-4 = 5 μM, Ir-1 = 5 μM, Rhodamine bisazide = 40 μM, NaAsc = 2 mM, ultrasound irradiation (1.0 W cm^{-2}, 1MHz, 50% duty cycle) in THF/H_2O under different conditions. I_0 = initial intensity of 525 nm. I = real-time intensity of 525 nm with various times under

ultrasound irradiation.

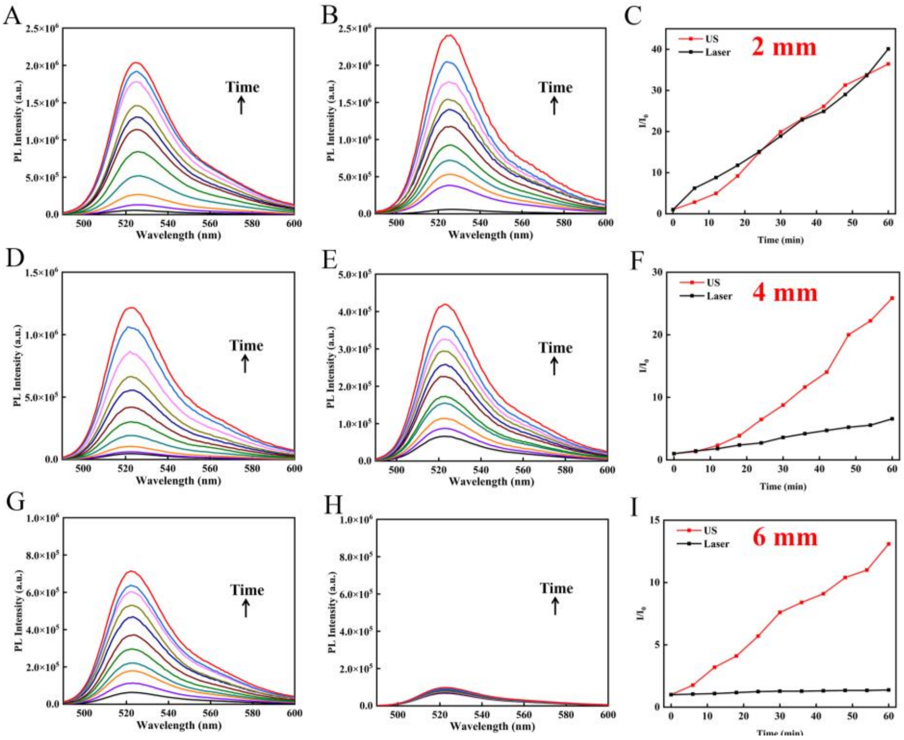

Figure S14. Fluorescence intensity of the reaction mixture of Ir-4 in aqueous solution using azide-caged Rhodamine 110 as the substrate upon ultrasound irradiation (1.0 W cm^{-2}, 1MHz, 50% duty cycle) at different time intervals covered with A) 2 mm chicken breast tissues, D) 4 mm chicken breast tissues, G) 6 mm chicken breast tissues. Fluorescence intensity of the reaction mixture under 635 nm laser irradiation (0.8 W cm^{-2}) at different time intervals covered with B) 2 mm chicken breast tissues, E) 4 mm chicken breast tissues, H) 6 mm chicken breast tissues. The change of PL intensity with the change of time under different conditions covered with C) 2 mm chicken breast tissues, F) 4 mm chicken breast tissues, I) 6 mm chicken breast tissues. I_0 = initial intensity of 525 nm. I = real-time intensity of 525 nm with various times under ultrasound irradiation.

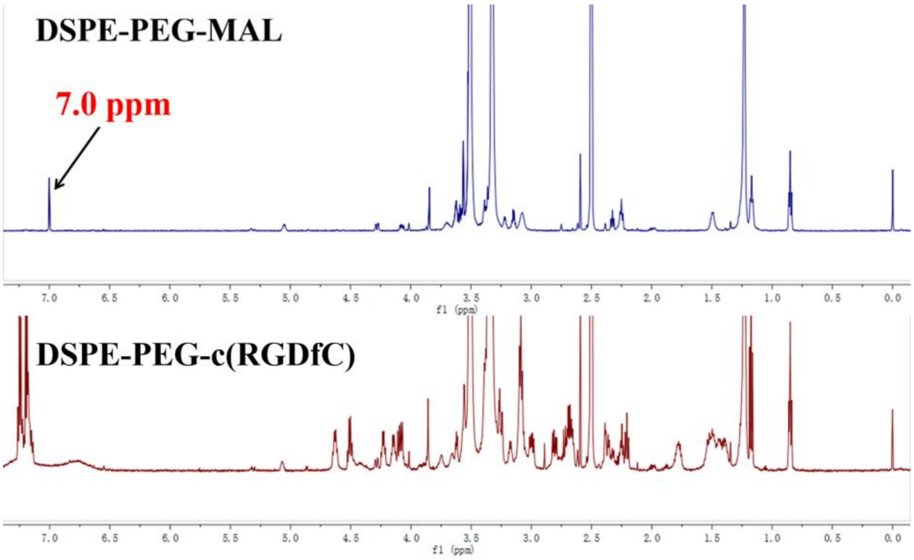

Figure S15. ^{1}H NMR spectra of DSPE-PEG-MAL and DSPE-PEG-c(RGDfC) (500 MHz, DMSO-d_6). The disappearance of the peak of the maleimide group at 7.0 ppm indicated the successful conjugation of c(RGDfC) to DSPE-PEG-MAL.

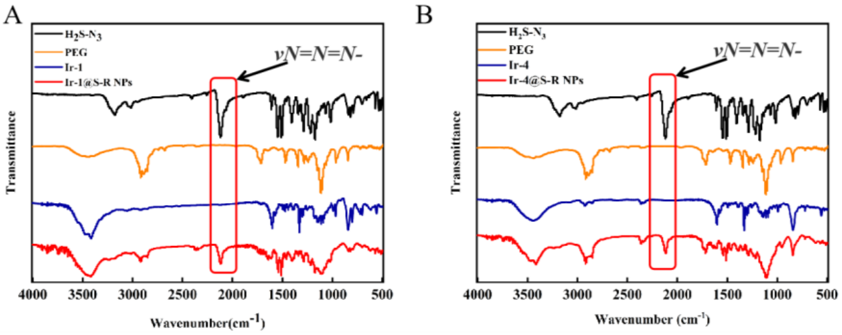

Figure S16. Fourier transform infrared (FT-IR) spectra of A) H_2S-N_3, PEG, Ir-1, Ir-1@S-R NPs. B) H_2S-N_3, PEG, Ir-4, Ir-4@S-R NPs.

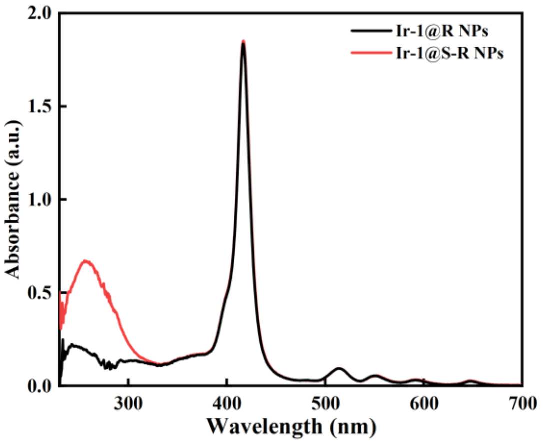

Figure S17. The absorption spectra of Ir-1@R NPs and Ir-1@S-R NPs at the same concentration in water.

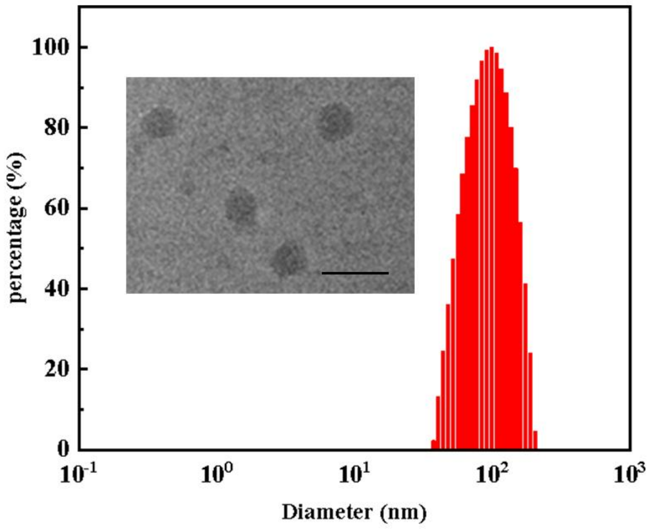

Figure S18. DLS diagram of Ir-1@S-R NPs, inset: the TEM images of Ir-1@S-R NPs. Scale bar: 150 μm.

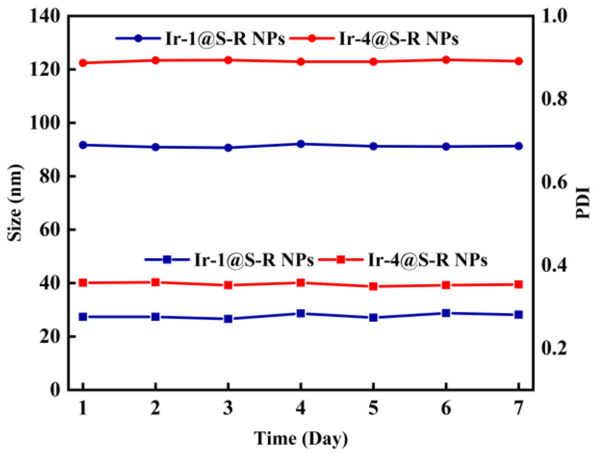

Figure S19. Size changes of different NPs in water during 7 days.

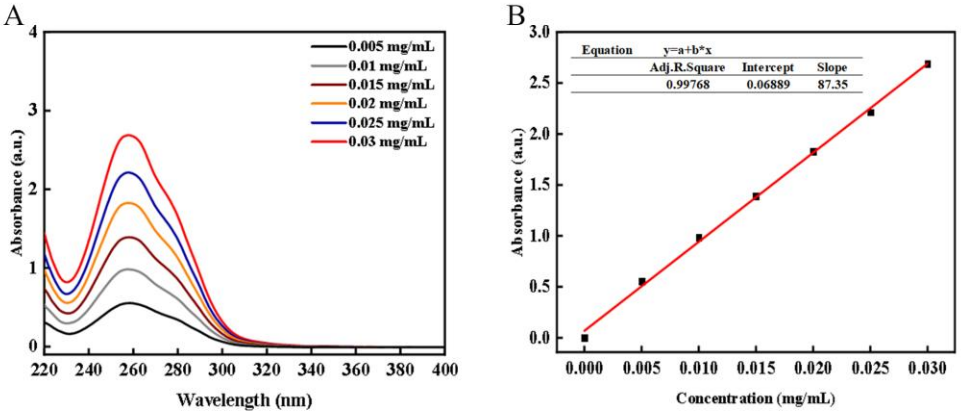

Figure S20. A) UV-vis absorption spectra of H_2S-N_3 solution at different concentrations in THF. B) The absorbance at 260 nm versus the concentration of H_2S-N_3.

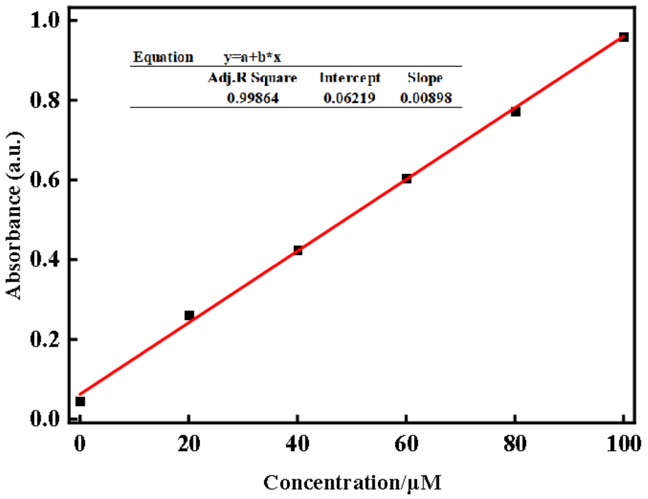

Figure S21. The H_2S calibration curve at 670 nm obtained with Na_2S using the methylene blue method.[5]

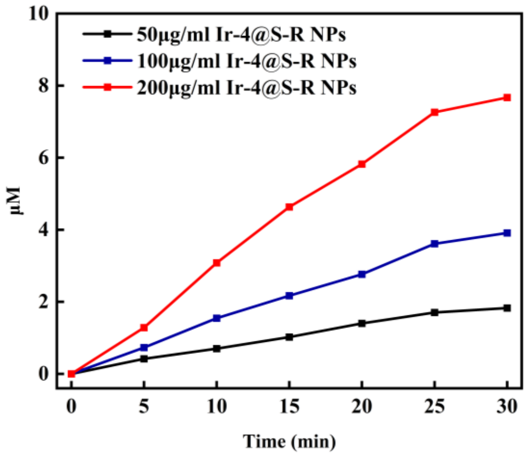

Figure S22. The amount of H_2S released from Ir-4@S-R NPs at concentrations of 50, 100 and 200 μg/ml in water under ultrasound irradiation (1.0 W cm^{-2}, 1MHz, 50% duty cycle).

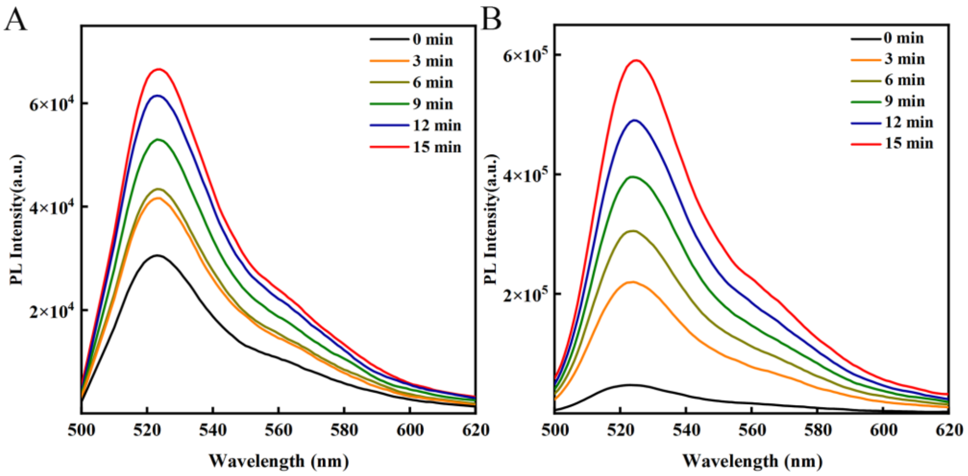

Figure S23. A) PL spectra changes of DCFH in the presence of PBS under ultrasound irradiation (1.0 W cm^{-2}, 1MHz, 50% duty cycle); B) PL spectra changes of DCFH in the presence of Ir-1@S-R NPs under ultrasound irradiation (1.0 W cm^{-2}, 1MHz, 50% duty cycle).

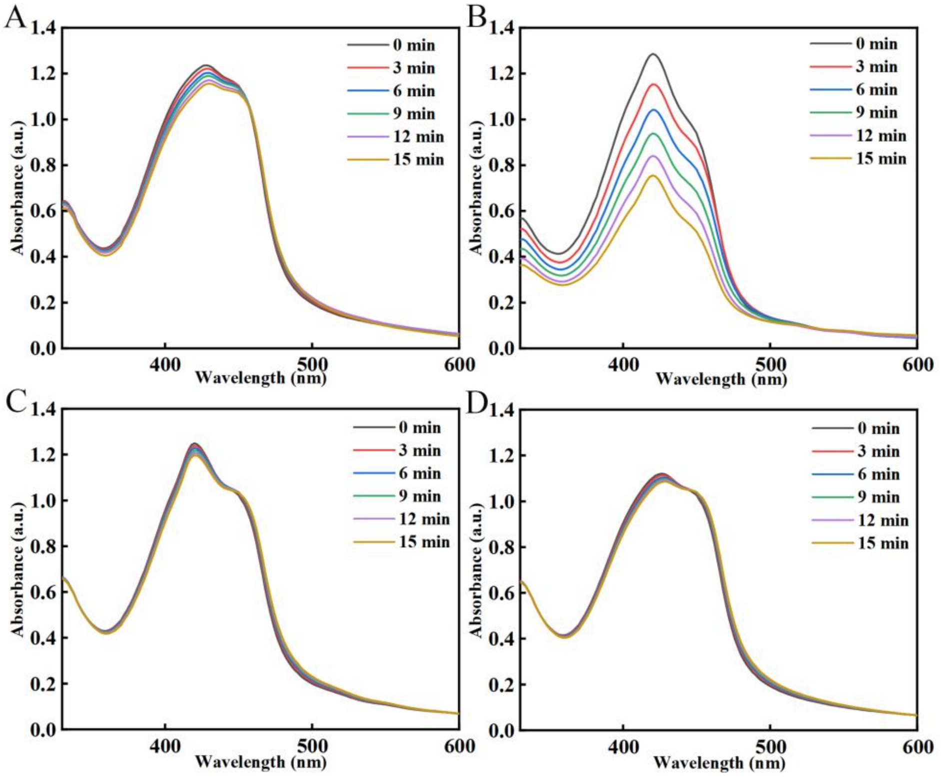

Figure S24. A) UV–vis absorption spectra changes of DPBF under ultrasound irradiation (1.0 W cm^{-2}, 1MHz, 50% duty cycle); B) UV–vis absorption spectra changes of DPBF in the presence of Ir-1@S-R NPs under ultrasound irradiation (1.0 W cm^{-2}, 1MHz, 50% duty cycle); C) UV–vis absorption spectra changes of DPBF in the presence of Ir-1@S-R NPs without ultrasound irradiation (1.0 W cm^{-2}, 1MHz, 50% duty cycle); D) UV–vis absorption spectra changes of DPBF in the presence of Ir-4@S-R NPs without ultrasound irradiation (1.0 W cm^{-2}, 1MHz, 50% duty cycle).

All spectra are in water.

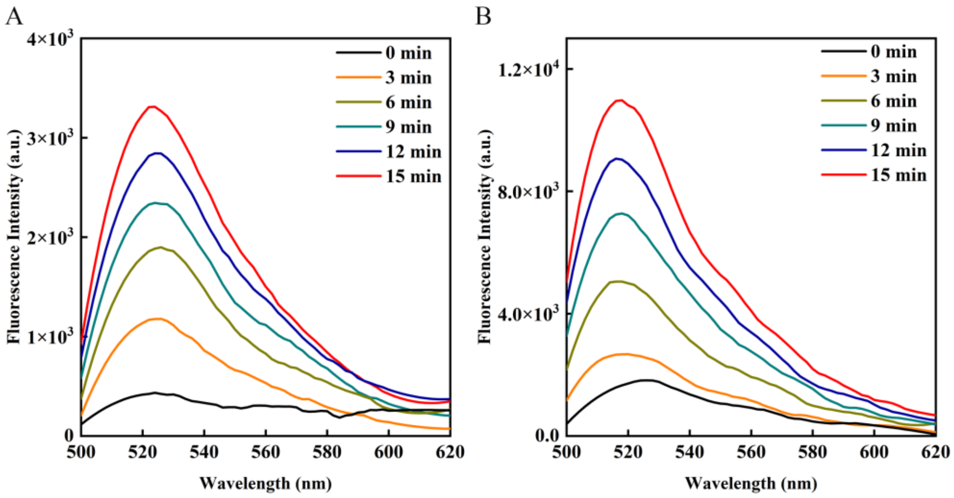

Figure S25. A) PL spectra of HPF in the presence of PBS under ultrasound irradiation (1.0 W cm^{-2}, 1MHz, 50% duty cycle); B) PL spectra changes of HPF in the presence of Ir-1@S-R NPs in water under ultrasound irradiation (1.0 W cm^{-2}, 1MHz, 50% duty cycle).

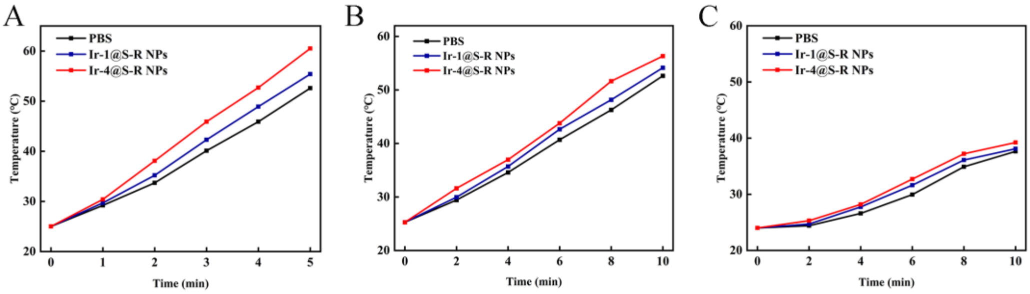

Figure S26. A) The acoustothermal heating curves of PBS, Ir-1@S-R NPs and Ir-4@S-R NPs under ultrasound irradiation (1.0 W cm^{-2}, 1MHz, 50% duty cycle) in 1 mL volume. B) The acoustothermal heating curves of PBS, Ir-1@S-R NPs and Ir-4@S-R NPs under ultrasound irradiation (1.0 W cm^{-2}, 1MHz, 50% duty cycle) in 3 mL volume. C) The acoustothermal heating curves of PBS, Ir-1@S-R NPs and Ir-4@S-R NPs under ultrasound irradiation (1.0 W cm^{-2}, 1MHz, 50% duty cycle) in 10 mL volume.

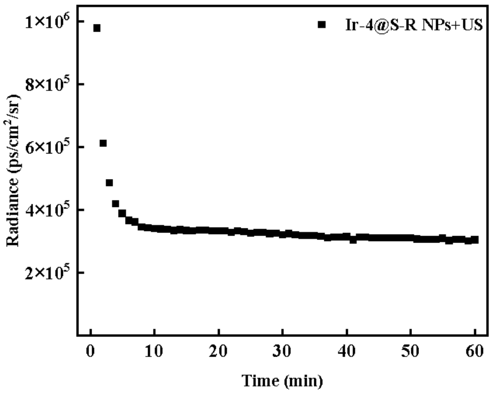

Figure S27. Decay of ultrasound-activated chemiluminescence intensities. Plot of persistent luminescence intensity of Ir-1@S-R NPs against the time post treatment with ultrasound.

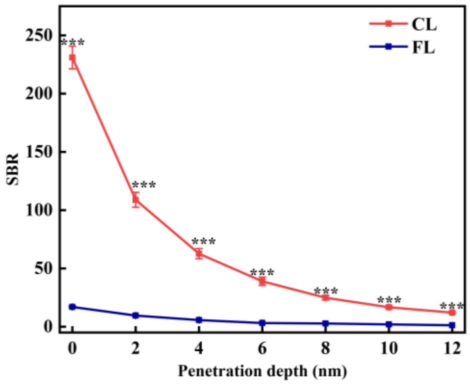

Figure S28. Signal-to-background ratios (SBRs) for CL or FL images as a function of chicken tissue depth. Data are represented as mean ± SD (n = 3). The data were analyzed by unpaired 2-tailed Student's t-test using GraphPad Prism 7. *p < 0.05, **p < 0.01, ***p < 0.001.

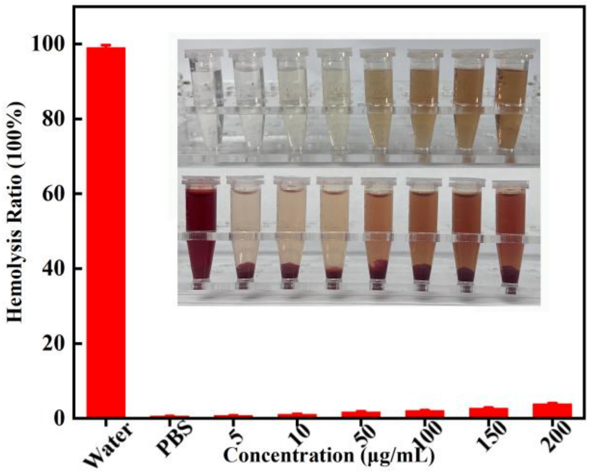

Figure S29. The hemolysis ratio of red blood cells treated with water and different concentrations of Ir-1@S-R NPs. Inset shows the solutions of red blood cells upon

different treatments (from left to right: water, PBS, 5, 10, 50, 100, 150, and 200 μg mL^{-1} of Ir-1@S-R NPs). Data are represented as mean ± SD (n = 3).

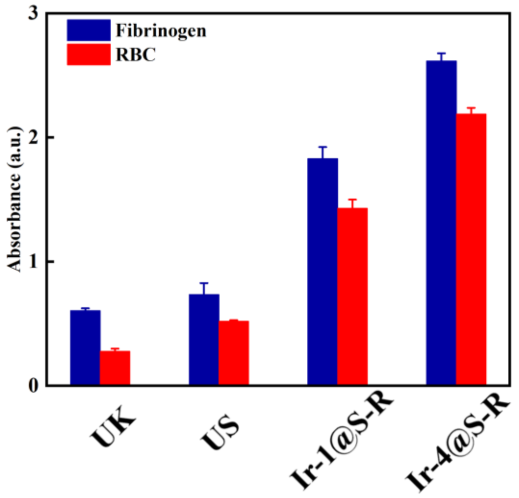

Figure S30. The absorbance at 450 nm (blue) and 540 nm (red) of the supernatant in different groups (UK, US, Ir-1@S-R + US, Ir-4@S-R + US) after 60 min of treatment. Data are represented as mean ± SD (n = 3).

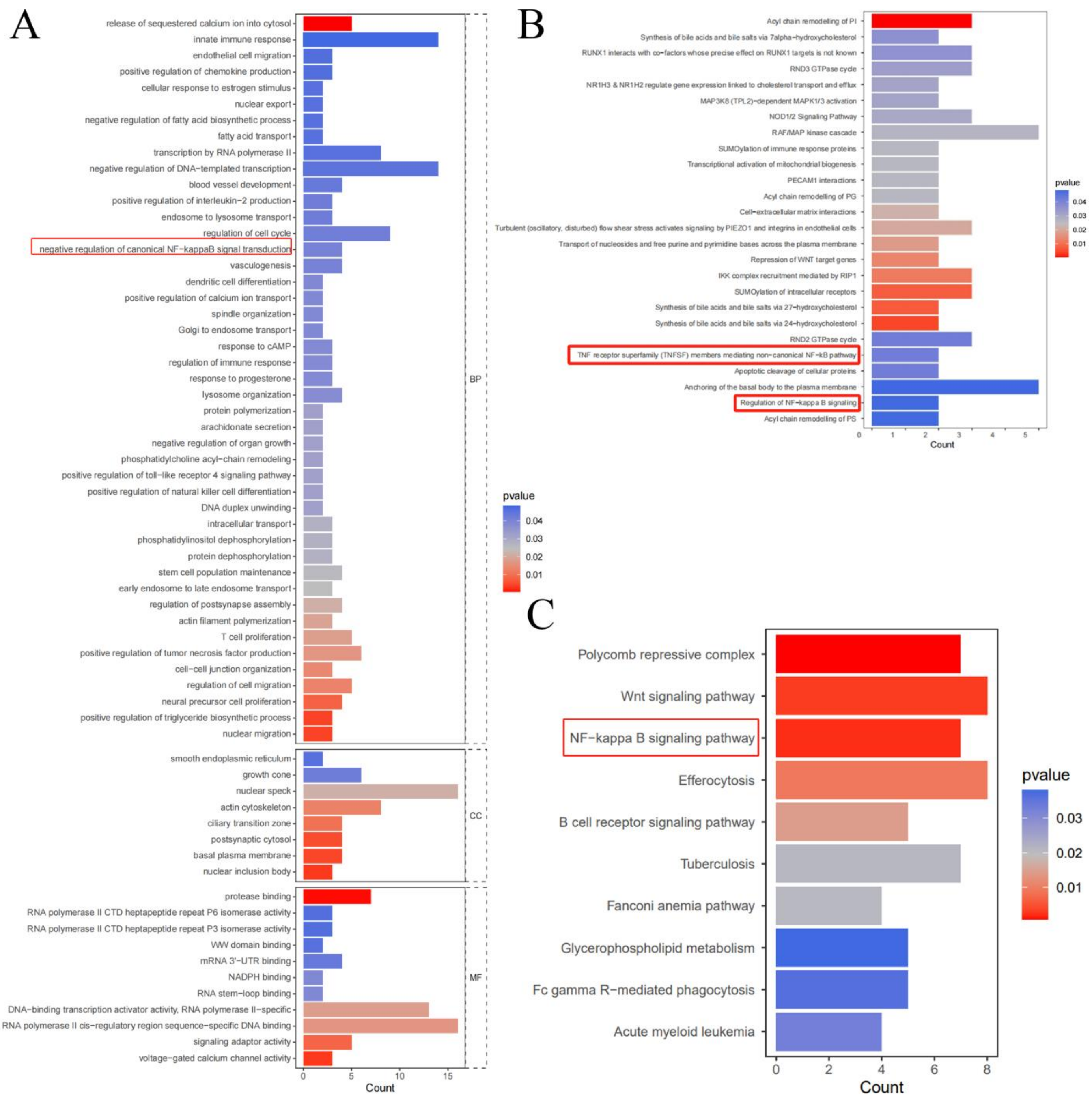

Figure S31. Figure S31 A) Gene ontology enrichment analysis of transcriptomics in the LPS group vs the treatment group; B) Reactome enrichment analysis of transcriptomics in the LPS group vs the treatment group; C) Kyoto encyclopedia of genes and genomes enrichment analysis of transcriptomics in the LPS group vs the treatment group.

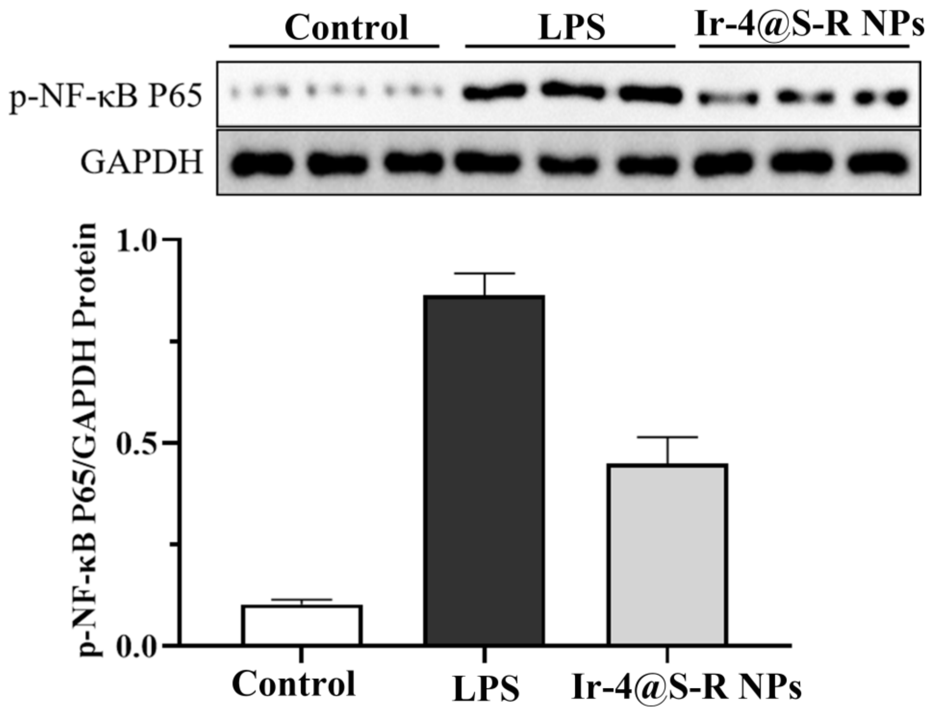

Figure S32. The Western blot experiment of control group, LPS group and Ir-4@S-R NPs group. Data are represented as mean ± SD (n = 3).

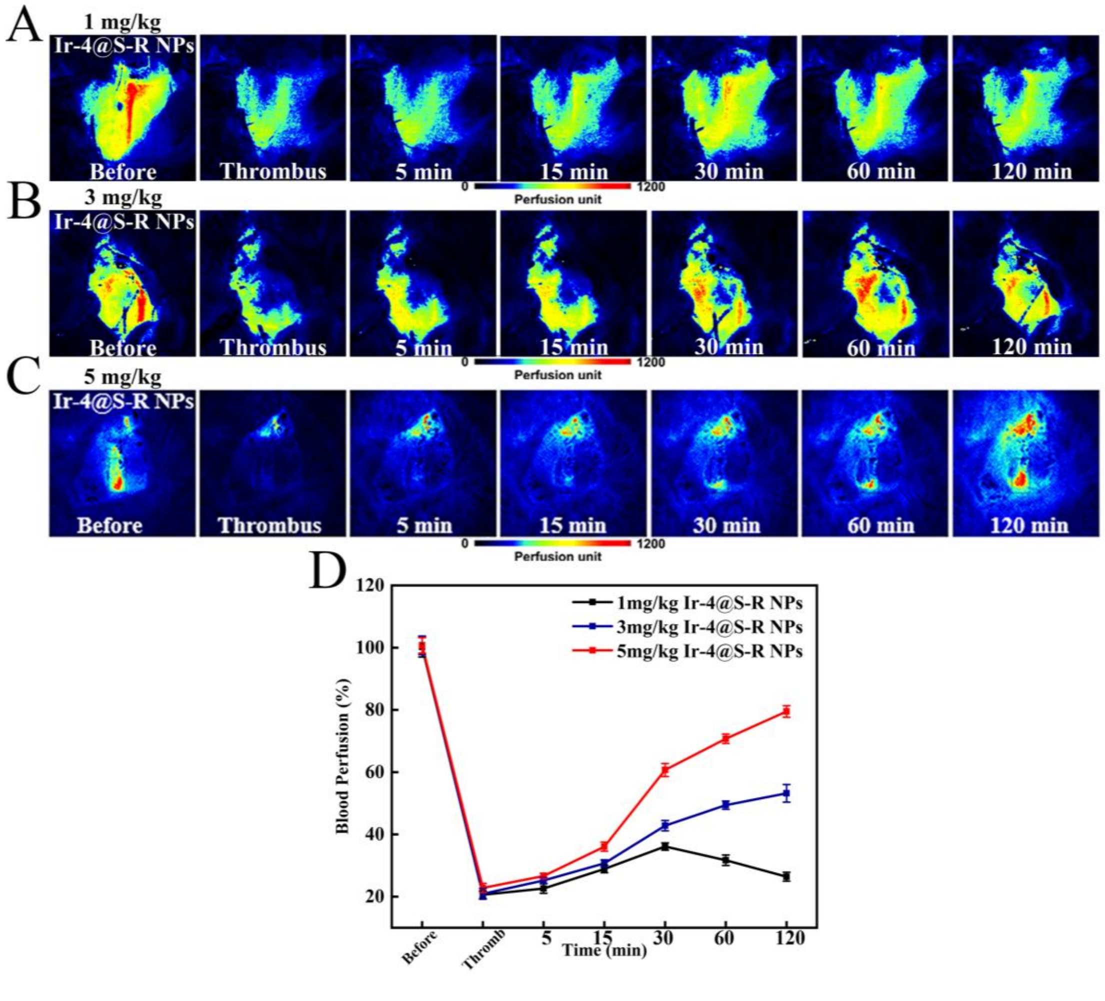

Figure S33. Representative LSBFMS analysis of the mouse carotid artery after $FeCl_3$ induction and different therapeutic treatments with A) 1 mg/kg, B) 3 mg/kg and C) 5 mg/kg Ir-4@S-R NPs + ultrasound; D) The corresponding relative blood perfusion of the mouse carotid artery after $FeCl_3$ induction and different therapeutic treatments with 1mg/kg, 3 mg/kg and 5 mg/kg Ir-4@S-R NPs + ultrasound. Data are represented as mean ± SD (n = 3).

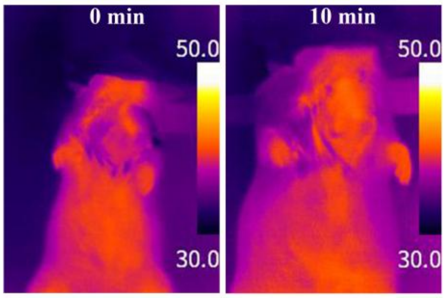

Figure S34. Infrared images a mouse treated with Ir-4@S-R NPs under ultrasound irradiation (1.0 W cm^{-2}, 1MHz, 50% duty cycle).

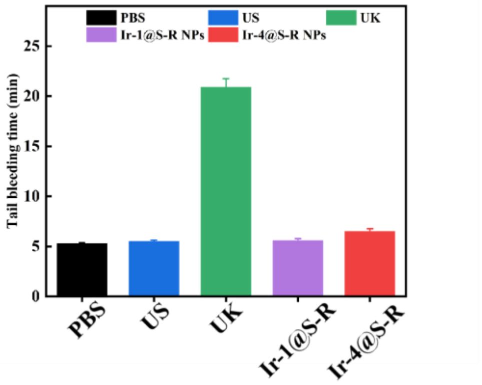

Figure S35. The tail bleeding time of mice after different treatments. Data are presented as mean ± SD (n = 3).

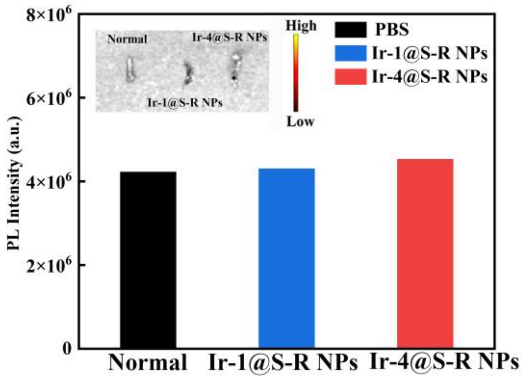

Figure S36. The fluorescence intensity of Evans blue permeability assays of carotid vessels in mice. Insets: the carotid vessels on an IVIS imaging system.

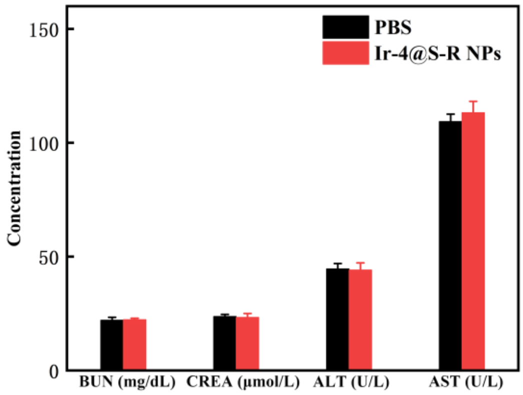

Figure S37. The hepatorenal indicator analysis of the mouse with different treatments. Data are represented as mean ± SD (n = 3).

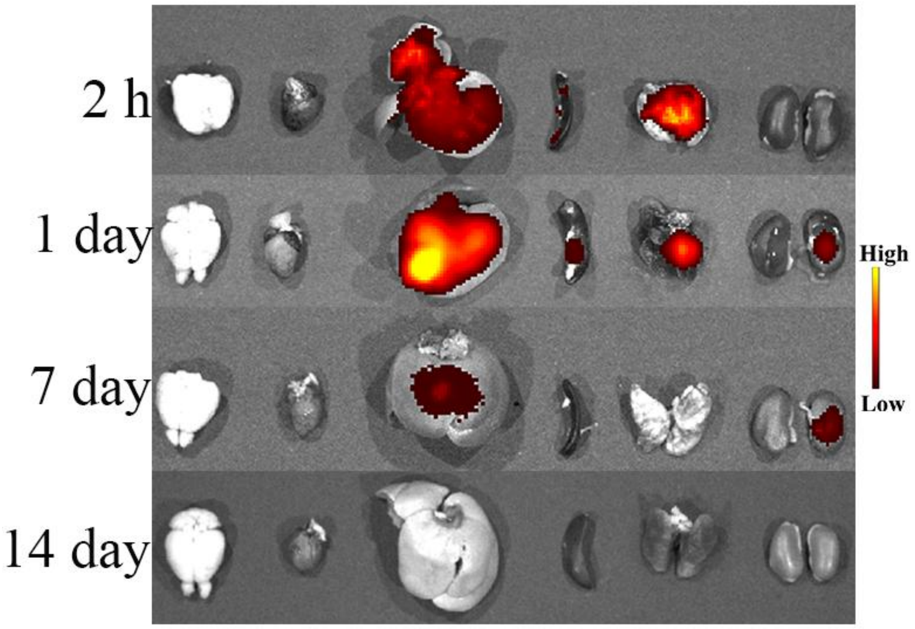

Figure S38. The fluorescence intensity of Ir-4@S-R NPs at different times in different mouse organs (left to right: brain, heart, liver, spleen, kidney and lung).

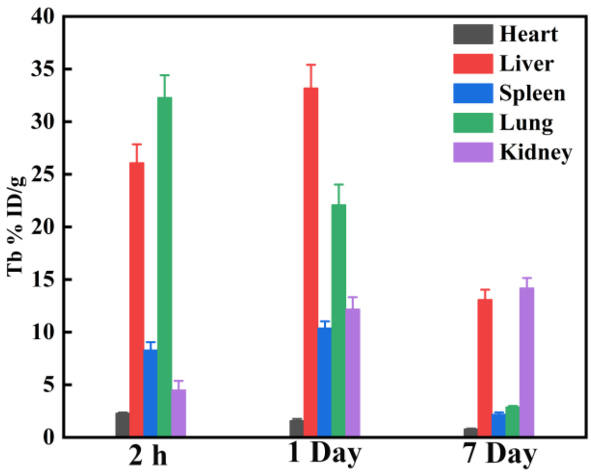

Figure S39. Evaluation of organ accumulation content of mice treated with Ir-4@S-R NPs by ICP-MS analysis. The contents are presented as mass percentage relative to injected dose per gram of organs (%ID/g). The Ir-4@S-R NPs were injected into the mouse through the tail vein and organs were collected after various periods of time. Data are represented as mean ± SD (n = 3).

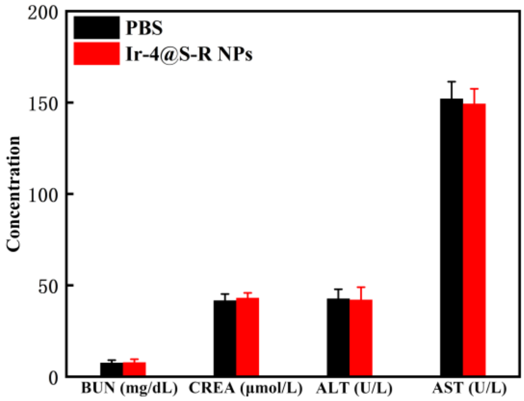

Figure S40. The hepatorenal indicator analysis of the rats with different treatments. Data are represented as mean ± SD (n = 3).

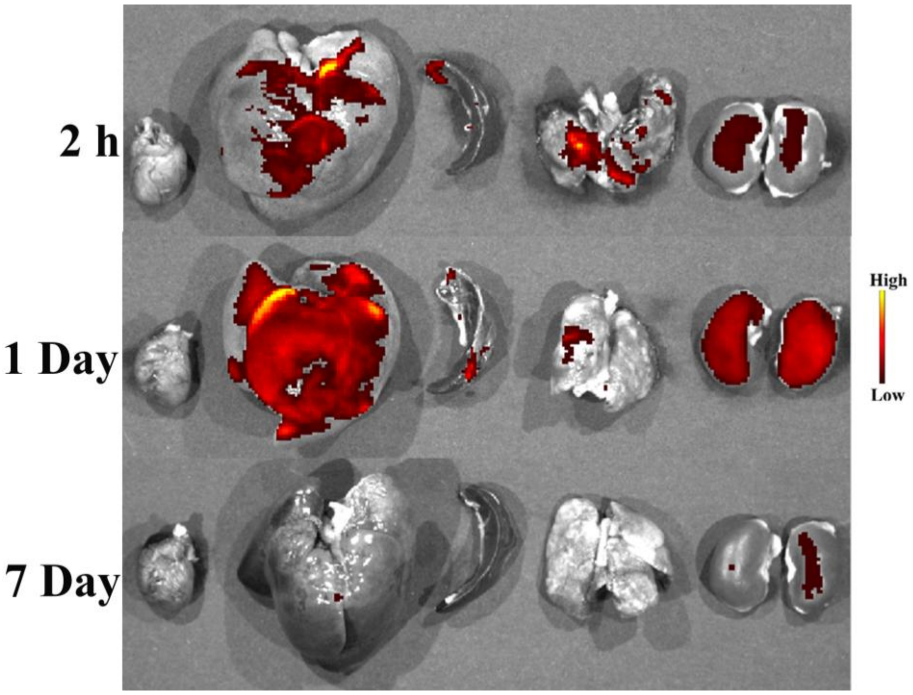

Figure S41. The fluorescence intensity of Ir-4@S-R NPs at different times in different rats' organs (left to right: heart, liver, spleen, kidney and lung).

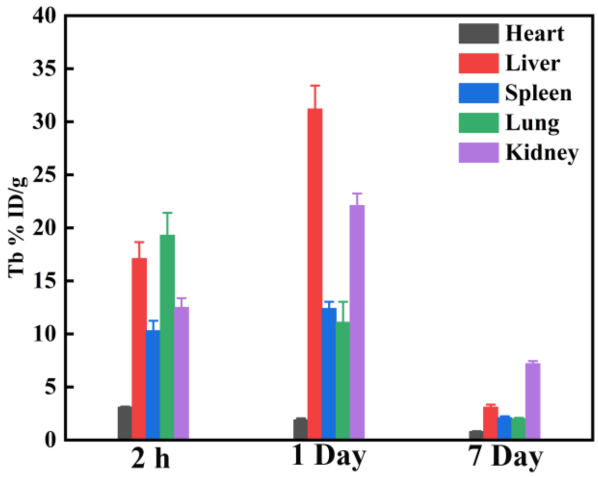

Figure S42. Evaluation of organ accumulation content of the rats treated with Ir-4@S-R NPs by ICP-MS analysis. The contents are presented as mass percentage relative to injected dose per gram of organs (%ID/g). The Ir-4@S-R NPs was injected into rats through the tail vein and organs were collected after various periods of time. Data are represented as mean ± SD (n = 3).

Table S1. Photophysical data of Ir-1 and Ir-4

	λ_{abs} (nm)	λ_{em} (nm)	Φ_p (%)
Ir-1[a]	244; 420; 518; 555; 594; 650	662; 718	2.31
Ir-1[b]	244; 416; 515; 550; 594; 648	656; 718	2.73
Ir-4[a]	247; 308; 426; 520; 560; 653	656; 720	1.77
Ir-4[b]	244; 308; 424; 516; 556; 652	656; 718	4.29

[a] Measured in THF solution (complex concentration = 1.0×10^{-5} M) at room temperature. [b] Measured in THF-H_2O mixtures (v:v = 1:9, complex concentration = 1.0×10^{-5} M) at room temperature.

[1] Z. Wang, L. Li, W. Wang, R. Wang, G. Li, H. Bian, D. Zhu, M. R. Bryce, *Dalton Transactions* **2023**, *52* (6), 1595-1601.
[2] Y. Quan, G. Lan, Y. Fan, W. Shi, E. You, W. Lin, *Journal of the American Chemical Society* **2020**, *142*, 1746−1751.
[3] P. K. Sasmal, S. Carregal-Romero, A. A. Han, C. N. Streu, Z. Lin, K. Namikawa, S. L. Elliott, R. W. Köster, W. J. Parak, E. Meggers, *ChemBioChem* **2012**, *13*, 1116-1120.
[4] M. Alajarin, M. Marin-Luna, M.-M. Ortin, P. Sanchez-Andrada, A. Vidal, *Tetrahedron* **2009**, *65*, 2579-2590.
[5] Y. Hu, X. Li, Y. Fang, W. Shi, X. Li, W. Chen, M. Xian, H. Ma, C*hemical Science* **2019**, *10*, 7690–7694.
